# Genomic recombination events may reveal the evolution of coronavirus and the origin of SARS-CoV-2

**DOI:** 10.1038/s41598-020-78703-6

**Published:** 2020-12-10

**Authors:** Zhenglin Zhu, Kaiwen Meng, Geng Meng

**Affiliations:** 1 School of Life Sciences, Chongqing University, No. 55 Daxuecheng South Road, Shapingba, Chongqing, 401331 China; 2 College of Veterinary Medicine, China Agricultural University, Beijing, 100094 China

**Keywords:** Computational biology and bioinformatics, Evolution

## Abstract

To trace the evolution of coronaviruses and reveal the possible origin of the severe acute respiratory syndrome coronavirus 2 (SARS-CoV-2), which causes the coronavirus disease 2019 (COVID-19), we collected and thoroughly analyzed 29,452 publicly available coronavirus genomes, including 26,312 genomes of SARS-CoV-2 strains. We observed coronavirus recombination events among different hosts including 3 independent recombination events with statistical significance between some isolates from humans, bats and pangolins. Consistent with previous records, we also detected putative recombination between strains similar or related to Bat-CoV-RaTG13 and Pangolin-CoV-2019. The putative recombination region is located inside the receptor-binding domain (RBD) of the spike glycoprotein (S protein), which may represent the origin of SARS-CoV-2. Population genetic analyses provide estimates suggesting that the putative introduced genetic sequence within the RBD is undergoing directional evolution. This may result in the adaptation of the virus to hosts. Unsurprisingly, we found that the putative recombination region in S protein was highly diverse among strains from bats. Bats harbor numerous coronavirus subclades that frequently participate in recombination events with human coronavirus. Therefore, bats may provide a pool of genetic diversity for the origin of SARS-CoV-2.

## Introduction

Since it was first identified in Wuhan, China^1–3^, severe acute respiratory syndrome coronavirus 2 (SARS-CoV-2) has become a global pandemic. To date, more than 16 million coronavirus disease 2019 (COVID-19) cases have been confirmed around the world. For the control and prevention of the disease, efforts have been made to trace the origin of SARS-CoV-2. Previous coronaviruses with outbreaks, such as severe acute respiratory syndrome (SARS) virus and Middle East respiratory syndrome (MERS) virus, originated from bats with an intermediate host^4,5^. In the publication of the first genome information for SARS-CoV-2, bats were also considered the original host of this virus^6^. Bat-CoV-RaTG13, a bat coronavirus isolated from *Rhinolophus affinis*, is 96% identical to SARS-CoV-2 at the whole-genome level^7^. Pangolin coronavirus was previously considered to have no direct relationship with SARS-CoV-2^8^, although viral communication was observed between Malayan pangolins (*Manis javanica*) and other hosts^9^. Later, a pangolin isolate, Pangolin-CoV-2019, was found to share only 91.02% identity at the whole-genome level with SARS-CoV-2, but showed higher sequence identity in the spike glycoprotein (S protein, 97.5%) coding sequence than Bat-CoV-RaTG13^10^. Therefore, the pangolin is considered a potential intermediate host of SARS-CoV-2^11–13^. It has been proposed that the receptor-binding domain (RBD) of the S protein in SARS-CoV-2 might be resulted from recombination between a virus similar or related to Bat-CoV-RaTG13 and a virus similar or related to Pangolin-CoV-2019^11,12,14,15^. The binding free energy between the SARS-CoV-2 RBD and human-ACE2 is significantly lower than that for SARS^16,17^, which partially explains the highly infectious activity of SARS-CoV-2. Thus, genomic recombination may be closely related to the pandemic of COVID-19 in human society. As a significant evolutionary mechanism, genetic recombination in RNA viruses forms novel chimeric genomes, driving the creation of viral diversity as well as the origin of novel viruses^18^. In-depth statistical analyses of genomic recombination among coronaviruses from different hosts, especially between pangolin coronaviruses and bat coronaviruses, should be important for tracing the origin of SARS-CoV-2 and may reveal interesting subsequent evolutionary patterns.

For the reasons described above, we scanned available documented coronavirus genomes^19–30^ and specifically examined possible recombination between SARS-CoV-2 and coronaviruses closely related to SARS-CoV-2 according to the coronavirus genomic phylogenetic tree^31^. To detect selection in recombinants, we performed population genetic analyses by calculating Pi, Tajima’s D and composite likelihood ratios (CLR) for 448 Coronaviridae samples and 26,312 SARS-CoV-2 samples. Our results revealed genomic recombination events between coronaviruses from different hosts and provided further evidence for the origin of SARS-CoV-2 via a recombination event between Bat-CoV-RaTG13 and Pangolin-CoV-2019 related strains^11,12,14^.

## Results

### Recombination between bat and pangolin coronaviruses may represent to the origin of SARS-CoV-2

**Figure 1:**
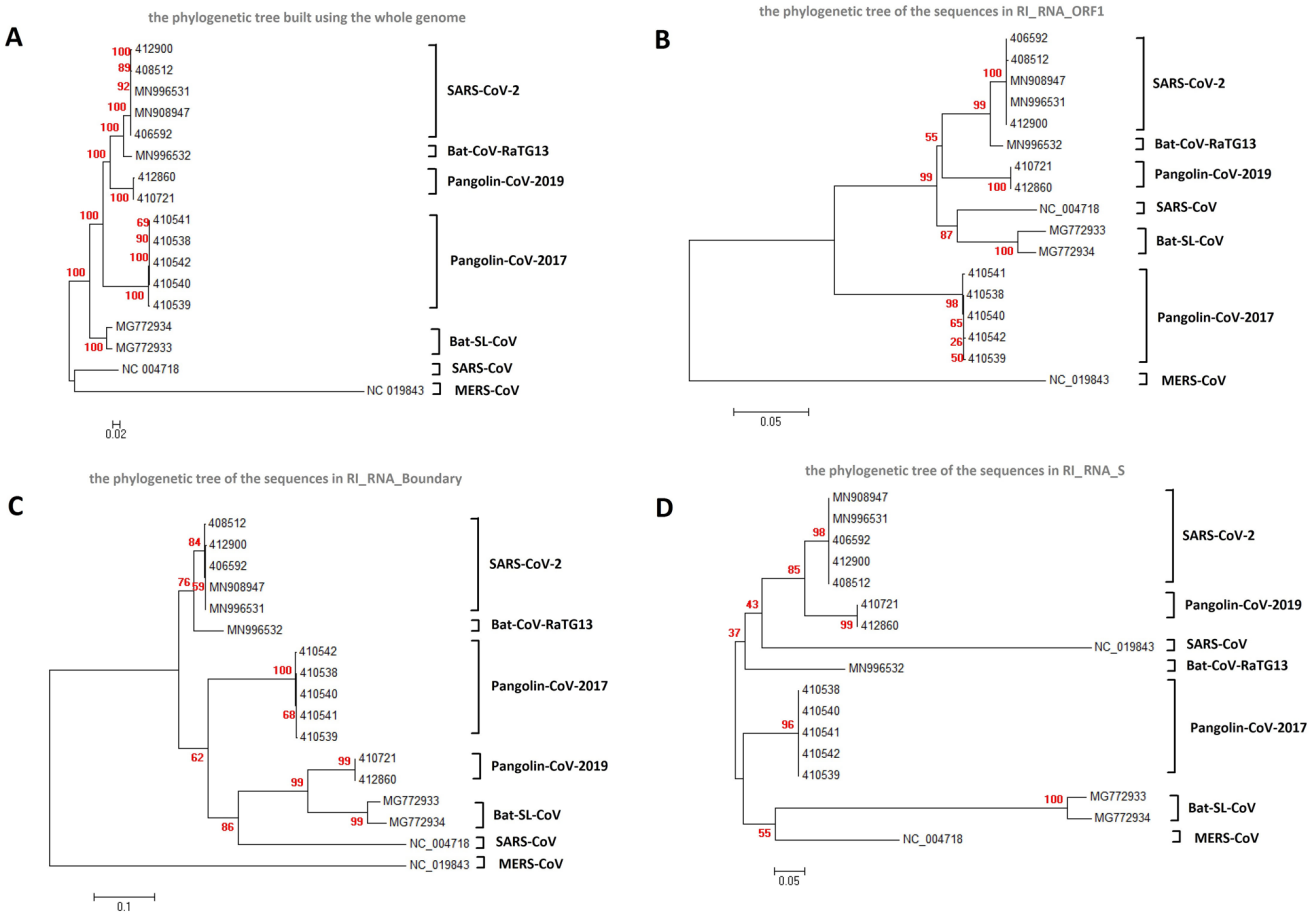
Verification of the three recombination events from phylogenetic trees. (**A**) Whole genome phylogenetic tree. (**B**) Phylogenetic tree built by sequences in RI_RNA_ORF1. (**C**) Phylogenetic tree built by sequences in RI_RNA_Boundary. (**D**) Phylogenetic tree built by sequences in RI_RNA_S. The trees were built using strains related to recombination and related outgroups. The names of the coronavirus to which the strains belong are listed to the right of the phylogenetic tree. The numbers marked in red are the marginal likelihoods of the tree. The trees were built by Mega using the Jukes-Cantor model. Phylogeny tests were performed using the bootstrap method with 5000 replicates.

We performed multiple sequence alignment for SARS-CoV-2 strains and proximal outgroups and identified 3 independent recombination events by RDP4, software to detect recombination^32^. Each event was supported by evidence from at least six statistical tests (requiring a *P*-value < 0.05 in each test) (Table 1). The phylogenetic tree of sequences in the recombination region was different from the phylogenetic tree built using the whole genome (Fig. 1). The three recombination events were also reflected in pairwise identity plots (Fig. S1). For further validation of the three recombination events, we also calculated pairwise genetic distances between coronaviruses, which were related to the three putative recombination events or outgroups. We performed calculations in the recombination region as well as the flanking sequences. The results (Table 2, Tables S1, S2) were consistent with the phylogenetic trees (Fig. 1) and pairwise identity plots (Fig. S1). For all three recombination events, the genetic distance, calculated for the recombination region, between the presumed recombinant and the presumed minor parent was the lowest among all the genetic distances between the putative recombinant and other outgroups. According to the definition in RDP4, the minor parent is the parental sequence that contributes the smaller fraction of the recombinant sequence, while the major parent is the parental sequence that contributes the larger fraction.

**Table 1:** Three putative recombination events between bat and pangolin coronaviruses.

Position		Major parent	Minor parent	Recombinant	Statistic tests (*P*-value)						
Start	End				RDP	GENECONV	Bootscan	Maxchi	Chimaera	SiSscan	3Seq
16,623	17,891	Some strains similar to Pangolin-CoV-2017 (410,541)	Some strains similar to Bat-SL-CoV (MG772933)	Some ancestral strain of SARS-CoV-2, Bat-CoV-RaTG13 and Pangolin-CoV-2019 (MN908947)	2.29E−13	1.43E−03	2.59E−11	3.82E−05	2.01E−06	1.26E−11	1.39E−08
21,187	22,368	Some strain similar to SARS-CoV-2 (MN908947)	Some strains similar to Bat-SL-CoV (MG772933)	Some strains similar to Pangolin-CoV-2019 (412,860)	6.20E−43	1.75E−12	6.52E−06	2.25E−14	7.05E−09	1.75E−10	1.26E−06
22,870	23,099	Some strains similar to Bat-CoV-RaTG13 (MN996532)	Some strains similar to Pangolin-CoV-2019 (412,860)	Some strains similar to SARS-CoV-2 (MN908947)	5.80E−14	1.83E−04	1.48E−04	5.02E−03	6.84E−04	NS	1.02E−11

‘Position’ refers to the start and end of the reference genome MN908947 (SARS-CoV-2). ’NS’ means not significant. The major parent and minor parent are the presumed parent contributing the larger fraction of the sequence and the presumed parent contributing the smaller fraction of the sequence, respectively. In cells, following the strain name, a representative strain ID is listed within a pair of small brackets. P-values based on seven statistical tests are also listed. Plots of alignments supporting these recombination events are shown in Fig. S1. Sequence IDs in brackets are exemplary sequences of the described strains.

**Table 2:** Estimates of evolutionary divergence between coronavirus sequences obtained using the Tajima-Nei model.

Species 1	Species 2	RI_RNA_S		5′ left (2000 bp)		3′ right (2000 bp)	
		Dist	Std. Err	Dist	Std. Err	Dist	Std. Err
SARS-CoV-2	Bat-CoV-RaTG13	*0.341	0.061	*0.061	0.006	*0.058	0.005
SARS-CoV-2	Pangolin-CoV-2019	*0.139	0.032	*0.247	0.014	*0.100	0.007
Bat-CoV-RaTG13	Pangolin-CoV-2019	*0.373	0.067	*0.247	0.014	*0.098	0.007
SARS-CoV-2	Pangolin-CoV-2017	0.264	0.050	0.184	0.012	0.151	0.010
Bat-CoV-RaTG13	Pangolin-CoV-2017	0.364	0.061	0.185	0.012	0.158	0.010
Pangolin-CoV-2019	Pangolin-CoV-2017	0.304	0.053	0.263	0.015	0.157	0.010
SARS-CoV-2	Bat-SL-CoV	0.766	0.119	0.295	0.015	0.199	0.012
Bat-CoV-RaTG13	Bat-SL-CoV	0.920	0.153	0.294	0.014	0.188	0.012
Pangolin-CoV-2019	Bat-SL-CoV	0.742	0.113	0.190	0.011	0.206	0.013
Pangolin-CoV-2017	Bat-SL-CoV	0.817	0.129	0.298	0.016	0.203	0.011
SARS-CoV-2	SARS-CoV	0.514	0.079	0.340	0.017	0.260	0.012
Bat-CoV-RaTG13	SARS-CoV	0.514	0.080	0.331	0.017	0.266	0.012
Pangolin-CoV-2019	SARS-CoV	0.519	0.076	0.355	0.018	0.271	0.013
Pangolin-CoV-2017	SARS-CoV	0.473	0.076	0.356	0.017	0.262	0.013
Bat-SL-CoV	SARS-CoV	0.970	0.169	0.381	0.017	0.236	0.012
SARS-CoV-2	MERS-CoV	1.341	0.366	0.786	0.038	0.806	0.035
Bat-CoV-RaTG13	MERS-CoV	1.277	0.324	0.772	0.037	0.810	0.035
Pangolin-CoV-2019	MERS-CoV	1.177	0.246	0.824	0.043	0.818	0.034
Pangolin-CoV-2017	MERS-CoV	1.304	0.282	0.817	0.038	0.830	0.036
Bat-SL-CoV	MERS-CoV	1.101	0.202	0.823	0.038	0.802	0.032
SARS-CoV	MERS-CoV	1.196	0.240	0.862	0.039	0.789	0.033

Analyses were performed on the sequences of RI_RNA_S (from 22,870 to 23,099 bp corresponding to MN908947), the 5′ left 2000 bp region (from 20,870 to 22,869 bp) and the 3′ right 2000 bp region (from 23,099 to 25,099 bp). The coronavirus genomes being compared are SARS-CoV-2 (MN908947), Bat-CoV-RaTG13 (MN996532), Pangolin-CoV-2019 (410,721), Pangolin-CoV-2017 (410,542), Bat-SL-CoV (MG772933), SARS-CoV (NC_004718) and MERS-CoV (NC_019843). ‘Dist.’ denotes genetic distance. ‘Std. Err’ denotes the standard error estimate(s). For convenience, we underlined the presumed recombinant (SARS-CoV-2). The values between the presumed recombinant and parents are marked by ‘*’.

**Figure 2:**
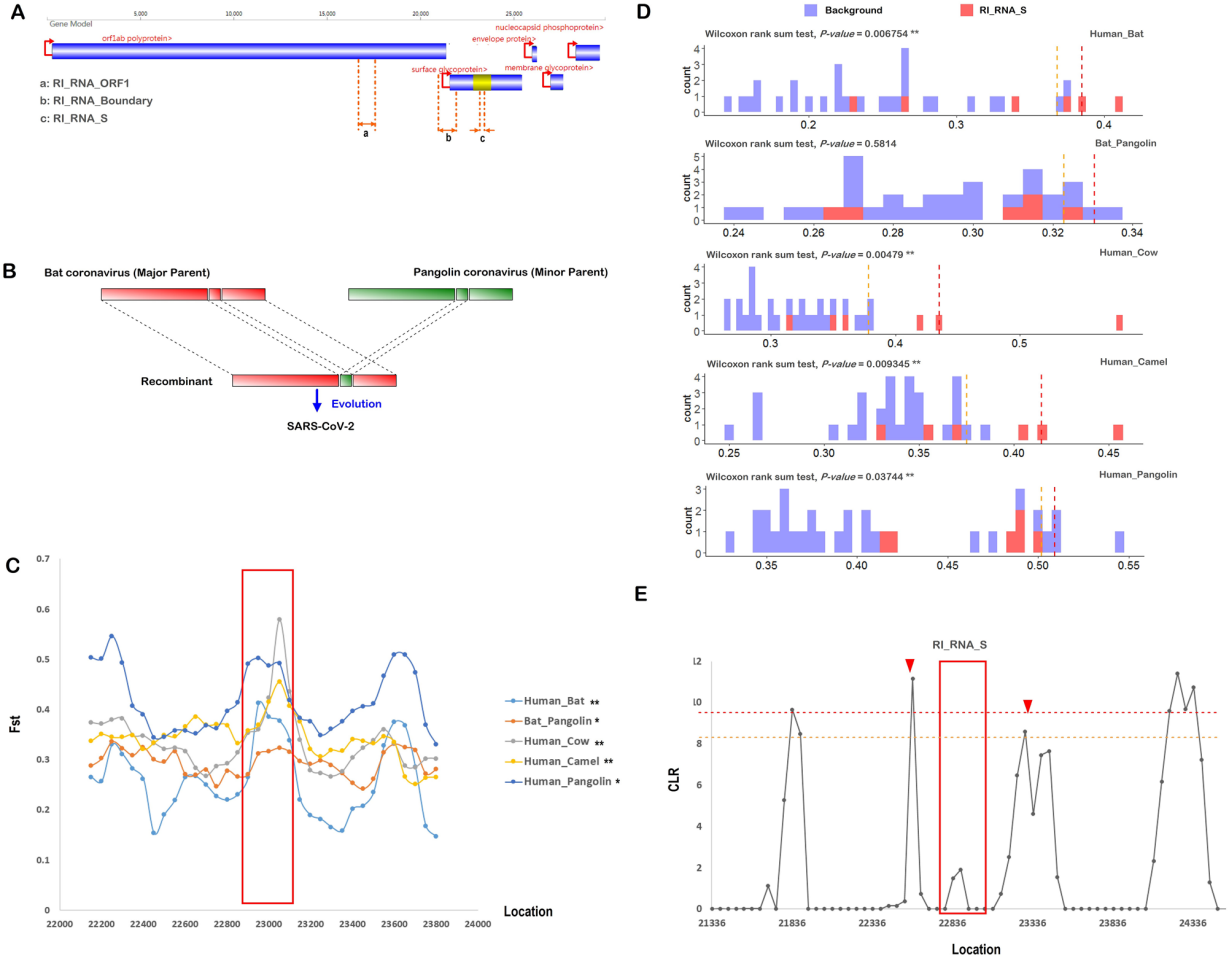
A sketch of the three recombination events and population genetic analysis results for RI_RNA_S. (**A**) Coordinate positions or positions of three recombinationally integrated RNA regions (indicated out by orange dotted lines) in the genome of SARS-CoV-2 (MN908947), with major proteins marked. ‘a’, ’b’ and ‘c’ refer to RI_RNA_ORF1, RI_RNA_Boundary and RI_RNA_S, respectively. Yellow represents the RBD in S protein. Red arrows with lines indicate the direction of transcription in SARS-CoV-2. (**B**) Diagram depicting a possible origin of SARS-CoV-2. (**C**) Snapshot of sliding window analysis of Fst (between coronaviruses from human and bat, human and pangolin, human and camel, human and cow as well as bat and pangolin). The region of RI_RNA_S is marked by a red rectangle. In the legend to the right, peaks at RI_RNA_S that are statistically significant (with values higher than the 0.05 threshold in the nearby region) are marked with ‘**’, and those with weak significance (with values higher than the 0.1 threshold in the nearby region) are marked with ‘*’. (**D**) Comparison of the distributions of Fst in RI_NA_S (red) and the nearby region (background, blue). Pairs of distributions in RI_NA_S and the flanking region were compared by the Wilcoxon rank sum test and a *P*-value is given. Vertical dashed lines denote the 0.05 cutoff (red) and 0.1 cutoff (orange) of the background distribution. (**E**) Sliding window analysis of CLRs with RI_RNA_S marked by a red rectangle. The result was generated using SARS-CoV-2 strains collected in April. Red triangles denote the two CLR peaks surrounding RI_RNA_S. The two peaks are significant or weakly significant if using the region nearby (from 21,000 to 25,000 bp) as a background, whose top 0.05 cutoff is denoted by a red dashed line and top 0.1 cutoff is denoted by an orange dashed line.

Two of the three potential recombination events may have altered the structures of two different pangolin-related coronavirus isolates, namely, an isolate possibly evolved from Pangolin-CoV-2017 and an isolate similar to Pangolin-CoV-2019. A 1260 bp fragment in some strains representing the ancestors of SARS-CoV-2, Bat-CoV-RaTG13 and Pangolin-CoV-2019 and a 1182 bp fragment in some strains similar to Pangolin-CoV-2017 may be recombinationally integrated sequences donated by bat isolates (bat-SL-CoVZC45 or bat-SL-CoVZXC21), suggesting that recombination between coronaviruses from bats and pangolins is not rare. One of these two recombinationally intergrated RNA fragments is located inside polyprotein 1ab (pp1ab, open reading frame 1 (ORF1)), referred to as RI_RNA_ORF1 in this manuscript, and the other fragment spans the 3′ end of ORF1 and the 5′ beginning of the S protein, referred to as RI_RNA_Boundary in this manuscript (Fig. 2A).

Our analysis confirmed that the 228 bp long sequence within the SARS-CoV-2 S protein (Fig. 2A) is likely to be an integrated sequence resulting from recombination between some strains similar to Bat-CoV-RaTG13 (NCBI accession No. MN996532) and some strains similar to Pangolin-CoV-2019 (NCBI accession No. MT121216; Table 1, Fig. 1D, Figs. S1C, S2). This recombination was significant in 6 independent statistical tests (Table 1). Moreover, we further validated of this recombination by performing sliding window analysis on sequence differences (Fig. S3) between SARS-CoV-2 and other coronaviruses proximal to SARS-CoV-2 in the phylogenetic tree (Fig. 1A). The recombination event was also validated by genetic distance analyses (Table 2). To reveal whether some other coronavirus strains contributed to the integrated sequence, we searched for recombination events using all reported coronavirus genomes (Table S3). We did not find any other recombination that may contribute to the 228 bp sequence. However, SARS-CoV-2 has not yet been isolated and identified from bats or pangolins. At the whole-genome level, Bat-CoV-RaTG13 shows higher identity with SARS-CoV-2 than Pangolin-CoV-2019. Our results suggested with high probability that SARS-CoV-2 originated from a bat coronavirus after recombinational integration of a RNA fragment from a pangolin coronavirus into the S protein gene (Fig. 2B). This putative integrated RNA fragment, referred to as RI_RNA_S in this manuscript, encodes a 76 AA long peptide and is located in the RBD (Fig. S2), which may influence the host preference of the virus. This recombination event may have played a key role in the origin of SARS-CoV-2.

### Evolutionary pattern of the putative recombinationally integrated fragment in the S protein

To understand the evolutionary role of the recombination that may have led to the origin of SARS-CoV-2, we performed sliding window analysis and genetic tests for coronavirus populations. We observed that RI_RNA_S has peaks of fixation index (Fst) values calculated between human and bat coronaviruses, between human and pangolin coronaviruses and between human and pangolin coronaviruses (Fig. 2C, Figs. S4, S5A). These Fst peaks have values higher than the 0.05 or 0.1 threshold when treating the nearby region as the background, indicating that they are significant or weakly significant (Fig. 2D, for specifics, see Materials and Methods). The significances of the human-bat and human-pangolin Fst peaks was also confirmed by comparing the distribution of the values inside RI_RNA_S and that in the flanking region (Fig. 2D). We also observed that Fst values between coronaviruses from other pairs of species mostly had peaks at RI_RNA_S (85.7%, 18/21, Fig. S5). Twelve of 21 of these peaks were confirmed by testing the difference in distribution between RI_RNA_S and the flanking region (Fig. 2D, Fig. S5B). The increase in differentiation reflected by the Fst peak suggests that RI_RNA_S is a featured segment. In other words, RI_RNA_S may be used to predict which host a coronavirus belongs to. RI_RNA_S may be important for coronavirus adaption to new hosts. Consistently, we observed a pair of CLR peaks adjacent to RI_RNA_S not only for SARS-CoV-2 strains collected in April (Fig. 2E) but also for those collected in March (Fig. S6). The two CLR peaks for April strains showed significance (threshold, 0.05) or weak significance (threshold, 0.1) in the nearby region. The two CLR peaks for March strains had values higher than the 0.05 threshold when treating the nearby region as the background, while one showed significance when considering the whole genome. These results suggested that the putative recombinationally integrated sequence in SARS-CoV-2 underwent adaptation.

We did not observe obvious fluctuations in the CLR or Tajima’s D within RI_RNA_S for any SARS-CoV-2 strains. One explanation for this result is that the RBD region is highly conserved in SARS-CoV-2. Compared with other human coronavirus, SARS-CoV exhibits a pair of CLR peaks adjacent to the corresponding region of RI_RNA_S (Fig. S4). The CLR peak of SARS-CoV near the 3′ right of RI_RNA_S is significant when considering the whole genome.

### Bats may provide a genomic pool for the origin of novel human coronavirus

**Figure 3:**
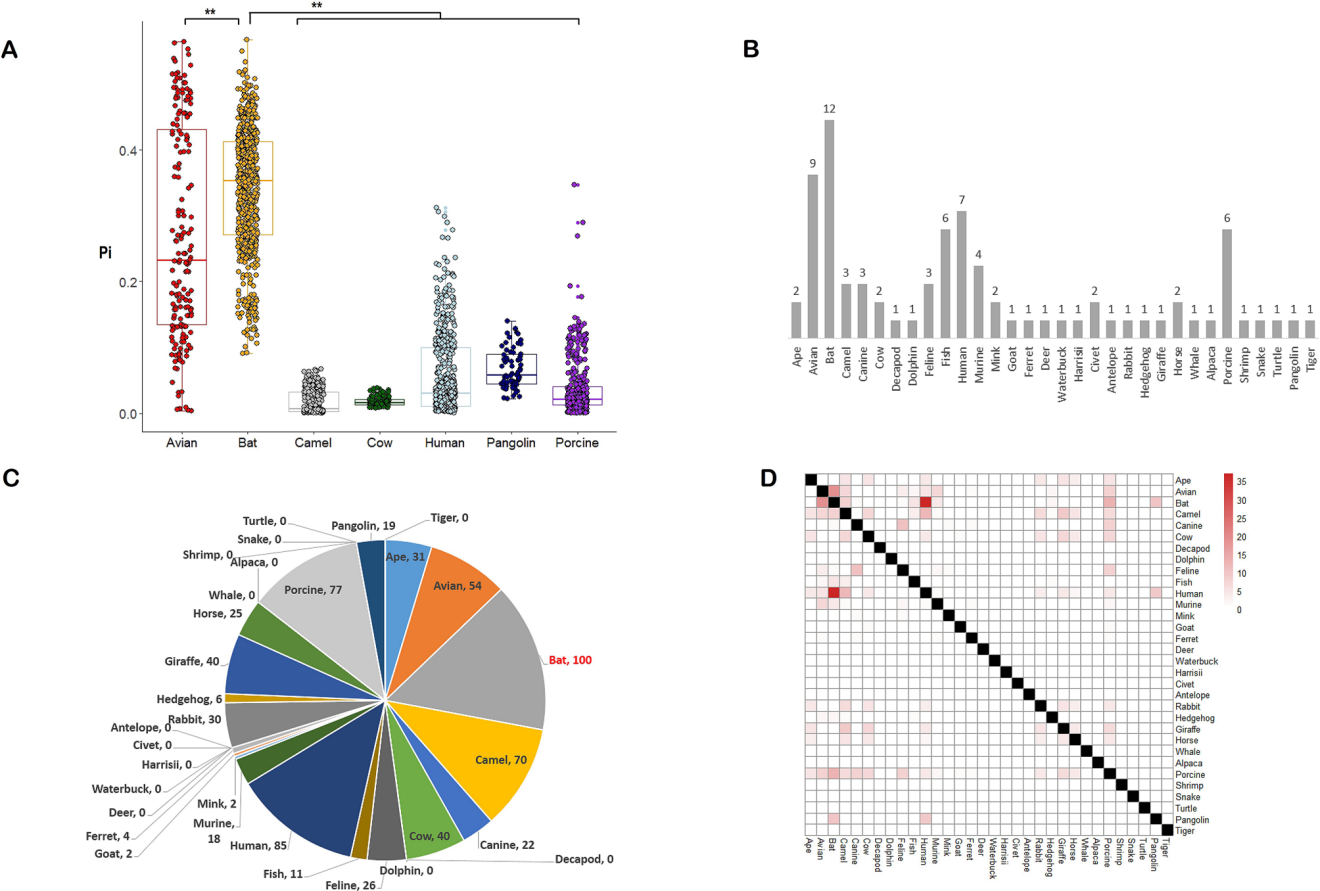
Evidence showing that bats may be a pool of genetic diversity. (**A**) Comparison of Pi in the nearby region of RI_RNA_S for coronaviruses from 7 different hosts, such as bat, human and pangolin. Pi values were calculated through a sliding window approach in the region from 21,500 to 25,000 bp according to MN908947. (**B**) Numbers of subclades of coronavirus in different hosts. (**C**) ie chart showing the numbers of independent recombination events in different hosts. Bat harbored the highest number and is marked in red. (**D**) Heatmap showing the numbers of independent recombination events occurring in coronaviruses between pairs of hosts the x and y axes). We did not consider recombination events between coronaviruses from the same host, which are marked by black squares.

There was a sharp decrease in RBD diversity (Pi) for human, camel and cow coronaviruses compared with bat coronaviruses. In contrast, for all coronaviruses isolated from bats, the Pi values in the RBD were high and there was a Pi peak at RI_RNA_S (Fig. S4). Considering that there are 12 reported clades of bat coronaviruses, population structure may contribute to the Pi peak. Therefore, we performed sliding window analyses on bat clades. We chose clades with more than 10 genome samples to ensure an adequate sample size. We found that RI_RNA_S has a Pi peak in 5 bat clades (Fig. S7A), and one shows significance (Rhinacovirus) within the region nearby. We performed the same analysis for 7 human clades. No clade had a Pi peak in RI_RNA_S with statistical significance (Fig. S7B). To avoid of the effects of population structure, we performed sliding window calculations of Pi in different clades. We calculated statistics to assess the differences between hosts. We found that bats had a higher Pi value for the nearby region of RI_RNA_S (from 21,500 to 25,000 bp) than other hosts (Fig. 3A). Bats also had a higher Pi for RI_RNA_S than other hosts (Fig. S8A). These findings highlight the high diversity of RBD sequences in coronavirus isolates from bats, which may provide a genetic pool for recombination that drives the evolution of coronaviruses in general and SARS-CoV-2 specifically.

Bat coronaviruses show a higher Tajima’s D than human coronaviruses (all coronaviruses isolated from humans, including SARS-CoV-2, SARS-CoV, MERS-CoV, and 229E-CoV) (*P*-value = 5.157e−08, Wilcoxon rank sum test) in the RBD region (Fig. S4). To test whether there is a difference in selection between bat and human coronaviruses, we slid the analysis window and calculated Tajima’s D, clade by clade, as we did for Pi above. We detected differences in Tajima’s D between bats and other hosts in RI_RNA_S and the nearby region of RI_RNA_S (Fig. S8B,C). Bat coronavirus did not show a deviation from neutrality (Tajima’s D = 0). However, there was no significant difference in Tajima’s D between bats and humans (Fig. S8B,C). Thus, the difference in Tajima’s D between bat and human coronaviruses in Fig. S4 may result from population structure.

Through a search in CoVdb^31^, we found that bat isolates had the highest number of subclades among 32 reported hosts (Fig. 3B), indicating that coronaviruses in bats may have differentiated at higher levels than those from other hosts because of population structure. Previous work indicated that most human coronaviruses originated from bats^33^. Interestingly, our analysis results show that bats rank first in terms of coronavirus recombination event quantity among 32 regular hosts (Fig. 3C). For recombination between coronaviruses from different hosts, the bat-human pair had the highest frequency (Fig. 3D, Fig. S9). A total of 43.5% (37/85) of human-related coronavirus recombination events were bat related. One hundred percent (10/10) of pangolin-related coronavirus recombination events were also bat related. The comparably high frequency of recombination between human and bat coronaviruses as well as between pangolin and bat coronaviruses (Fig. S9) may explain the origin of SARS-CoV-2.

### Recombination between bat and pangolin coronaviruses

It is likely that Pangolin-CoV-2017 or Pangolin-CoV-2019 contributed to the origin of SARS-CoV-2, although the two recombination events referred in this work showed no direct contribution to the origin of SARS-CoV-2. The putative integrated sequence from Bat-SL-CoV in Pangolin-CoV-2019 (Figs. 1, 2A) may have made the viral genome less similar to SARS-CoV-2. Nevertheless, recombination among coronaviruses between bats and pangolins may have generated other novel strains that can be transmitted between species. SARS-CoV-2 may be just a recent example. We observed a CLR peak at RI_RNA_ORF1 for SARS-CoV-2 strains (Fig. S10). The CLR peak was significant in the nearby region and confirmed by testing the distribution differences (*P*-value = 9.997e−08). The CLR peaks were re-evaluated and confirmed using all SARS-CoV-2 strains collected in March (Fig. S11A). We observed a nonsignificant peak for those in April (Fig. S11B). RI_RNA_Boundary showed a peak in the CLR calculated using human isolates (Fig. S12). The values in the peak were significantly higher than those in the flanking region (extended by 1000 bp, *P*-value = 7.375e−06). We also observed a peak in the CLR calculated using SARS-CoV-2 strains collected in April (Fig. S11D). We observed peaks in the CLR calculated based on SARS-CoV-2 strains collected in March (Fig. S11C), but these peaks were not significant. There was a peak in Fst (significant in nearby region, threshold, 0.05) calculated between human and bat coronaviruses at RI_RNA_Boundary (Fig. S12), which was also confirmed by comparing the distributions in RI_RNA_Boundary and the flanking region (P-value = 7.11e−05). The same was observed for Fst values between coronaviruses from most other pairs of species at RI_RNA_Boundary (76.2%, 16/21, Fig. S13). Nine of the 16 peaks of Fst values showing local significance were also confirmed by testing the distribution (Table S4). These results indicated that these putative recombinants were evolutionarily active regions.

## Discussion

Previous efforts were made to detect evidence of recombination, but did not support a relationship between recombination and the origin of SARS-CoV-2^1–3^. In contrast, other studies revealed that recombination could be associated with the origin of COVID-19^15^. In relation to this issue, our observations indicate that SARS-CoV-2 possibly originated from recombination between bat and pangolin coronaviruses. We reached this conclusion through comprehensive analyses of all reported coronavirus genomes from different hosts. Important evidence was provided by genomic sequence analysis of Pangolin-CoV-2019 (412,860) and Bat-CoV-RaTG13 (MN996532).

The putative recombinationally integrated sequence provided by some strains similar to Pangolin-CoV-2019 was located inside the RBD of the S protein region. Our analyses indicated that RI_RNA_S is under positive selection in SARS-CoV-2 populations. These results supported the evolutionary importance of RI_RNA_S. However, more experiments are needed to understand whether and how RI_RNA_S functions differently in SARS-CoV-2 and Bat-CoV-RaTG13.

Unlike human coronavirus genomes, coronavirus genomes isolated from other hosts are limited in terms of public availability. To overcome this, we pooled all coronaviruses from different subclades and collection times that were isolated from the same host. Although such pooling can inflate genetic diversity levels, we used the same pipeline for all coronavirus strains to reduce deviation from reality. Bat coronaviruses had a higher Tajima’s D than human coronaviruses. Considering that the number of bat samples was smaller (176) than the number of human samples (972), the low Tajima’s D for human coronaviruses may have been caused by sample size differences, as the larger the sample size is, the more negative Tajima’s D might be. The negative Tajima’s D for human coronaviruses was located inside a negative valley, as shown in Fig. S4. Thus, it is hard to infer whether sample size led to the difference in Tajima’s D between human and bat coronaviruses.

Our analyses provide further support that SARS-CoV-2 originated from bats, considering that bat isolates may be the major parent contributing the largest fraction of sequences. However, we still cannot conclude that SARS-CoV-2 originated from bats because of the lack of direct evidence. There is high genetic diversity in the S-protein of coronavirus strains from bats, but not in strains from other hosts, suggesting that bats are a reservoir of genetic diversity upon which natural selection can act. Compared to other hosts, bats also have more coronavirus subclades in terms of taxonomy. Bat coronaviruses may have more chances to take part in recombination than coronaviruses from other hosts and thus play the most important role in the origin and recombination of human coronaviruses among all known coronavirus hosts. Thus, avoiding contact with wild bats should be important for preventing future coronavirus associated pandemic diseases in humans.

## Methods

### Identification of recombination events

We collected genomic sequences of coronaviruses from NCBI, GISAID (http://www.gisaid.org) and CoVdb^31^. We collected 3140 non-SARS-CoV-2 coronavirus (Table S5) and 26,312 SARS-CoV-2 strains (Table S6). Using all coronavirus strains, we performed whole-genome alignments by CUDA ClustalW^34^ and built a phylogenetic tree, according to which we observed that bat- and pangolin-isolated strains were proximal to SARS-CoV-2. We chose SARS-CoV-2, severe acute respiratory syndrome coronavirus (SARS-CoV), Middle East respiratory syndrome coronavirus (MERS-CoV) and strains whose hosts are bats or pangolins, and then performed recombination detection by RDP4^32^, which used RDP (the algorithm used to test for recombinants in RDP4 software)^35^, GENECONV^36^, Bootscan^37^, Maxchi^38^, Chimaera^39^, SiSscan^40^ and 3Seq^41^ as statistical test methods for recombinants. We chose to perform a full exploratory scan using all methods in the software. In this way, we identified three putative recombination events between bat and pangolin coronaviruses. We used MEGA^42^ to perform local alignments, to build maximum likelihood trees by the Jukes-Cantor model^43^ and to test the phylogeny by 5000 bootstrap replicates. For further verification of the recombination detected, we estimated the evolutionary divergence between coronavirus sequences using the Tajima-Nei model^44^ by Mega^42^. We chose one strain in each coronavirus as a target. We performed analyses on RI_RNA_S, RI_RNA_ORF1, RI_RNA_Boundary and the nearby 2000 bp sequences of these fragments. We also wrote Perl scripts to perform sliding window calculations of the nucleotide differences between SARS-CoV-2 and other coronaviruses proximal to SARS-CoV-2 in the phylogenetic tree shown in Fig. 1.

To obtain an overall and general view of possible recombination events within all reported coronaviruses, we retrieved all coronavirus genomes from CoVdb^31^ and then filtered out unique genomic sequences using CD-HIT^45^, requiring an identity > 95% and a coverage > 95% to speed up postanalyses. With these unique genomic sequences, we performed whole-genome sequence alignment and used RDP4^32^ to search for possible recombination events. We also performed a full exploratory scan using all methods provided in RDP4. In this way, we identified 1149 putative recombination events. We discarded the cases in which the recombination signal could have been caused by an evolutionary process other than recombination. With these identifications procedures combined, we identified 532 putative independent recombination events (Table S3).

### Annotation of coronavirus strains

We annotated these recombination events based on the information provided by CoVdb^31^, where the classification of coronaviruses was first based on NCBI taxonomy. The properties of unclassified strains were identified by searching against a manually curated reference set, which include representative sequences of subclades. Other information, such as the host and collection region, was retrieved from the NCBI or GISAID and then curated manually.

### Population genetic analyses

We used the online tools in CoVdb^31^ to perform population genetic analysis. In the platform, coronavirus strains isolated from the same host are grouped together. For viruses isolated from humans, those related to the same disease are also grouped together. As a result, there were 173, 216, 38 and 21 samples for SARS-CoV-2, SARS-CoV, HKU1-CoV and TGEV, respectively (for details, see the information on coronavirus strains at http://covdb.popgenetics.net/v2). Because of the limited data, we performed analysis of all coronaviruses isolated from the same host without considering the collection date or subclade. As a result, there were 972, 176, 303, 34 and 90 samples for human, bat, camel, cow and murine coronaviruses, respectively. Pi^46^ and Tajima’s D^47^ were calculated by VariScan 2.0^48,49^. The CLR^50,51^ was calculated by SweepFinder2^52^. We performed multiple sequence alignment of the first 19 sequenced coronavirus genomes and obtained a consensus sequence as the ancestral state of the SARS-CoV-2 genome. Based on the ancestral genome, we polarized the alleles to define the ancestral and derived alleles. We considered all other windows except the one tested as the background neutral site frequency spectrum (SFS). We ranked all CLR values from sliding window analysis along the genome and set the top 5% as the cutoff for filtering out significant points in the whole genome. The genomic sequences of SARS-CoV-2 collected in March (16,270 samples) and April (10,042 samples) were downloaded from GISAID. Tests were performed for both March and April to evaluate whether some selection signatures had changed with time. Moreover, more SARS-CoV-2 samples were collected in March and April than in January (344 samples) and February (661 samples).

### Statistics

We calculated the statistics referred to in this work by R. A heatmap was created by the R package “pheatmap” and TBTools^53^. Most data extraction and clustering work was performed by writing Perl pipelines. When calculating statistics for all coronavirus recombination events shown in Fig. 3D and Fig. S9, we did not differentiate which was the recombinant, major parent or minor parent. They were all considered to take part in or related to recombination. To test the significance of a peak of a population genetic track after sliding window analysis, we extended the target region by a distance of nearly 1000 bp to the left and right and used the extended region as the background. We tried to identify where the values of the target region were located in the distribution of the background, as shown in Fig. 2D. If the maximum value of a peak was higher than the 0.05 threshold, the peak inside the target region was considered to be significant in the region nearby, or locally significant. If it was higher than the 0.1 threshold, the peak was considered weakly significant. We also performed a Wilcoxon rank sum test between the distribution of track values in the target region and that in the flanking region, including the 1000 bp sequences to the left and right, to validate the significance of the peak. Moreover, we tried to identify the position of the peak value in the distribution of all the values in the whole genome. If a peak was inside the top 5% section (*P *value < 0.05), it was considered to have whole-genome significance.

## Supplementary Information

Supplementary Information 1.

Supplementary Information 2.
