# Supplementary material for: Genomic recombination events may reveal the evolution of coronavirus and the origin of SARS-CoV-2
Source: Sci Rep. 2020 Dec 10;10:21617. doi: 10.1038/s41598-020-78703-6 (PMC7728743; doi:10.1038/s41598-020-78703-6)
Supplement: Supplementary file 1 — Supplementary Information 1. [file 41598_2020_78703_MOESM1_ESM.docx]

Genomic recombination events may reveal the evolution of coronavirus and the origin of SARS-CoV-2

Zhenglin Zhu^1*^, Kaiwen Meng^2^, Geng Meng^2*^

1. School of Life Sciences, Chongqing University, Chongqing, China

2. College of Veterinary Medicine, China Agricultural University, Beijing, China

^*^Corresponding authors

Zhenglin Zhu, School of Life Sciences, Chongqing University, No.55 Daxuecheng South Road, Shapingba, Chongqing, 401331, China.

TEL: (86)23-6512-2686, FAX: (86)23-6512-2689, zhuzl@cqu.edu.cn

Geng Meng, College of Veterinary Medicine, China Agricultural University, Beijing, 100094 China

TEL: (86)10-6273-3466, FAX: (86)10-6273-3466, mg@cau.edu.cn

Keywords: SARS-CoV-2; COVID-19; recombination; origination; coronavirus;

**A**


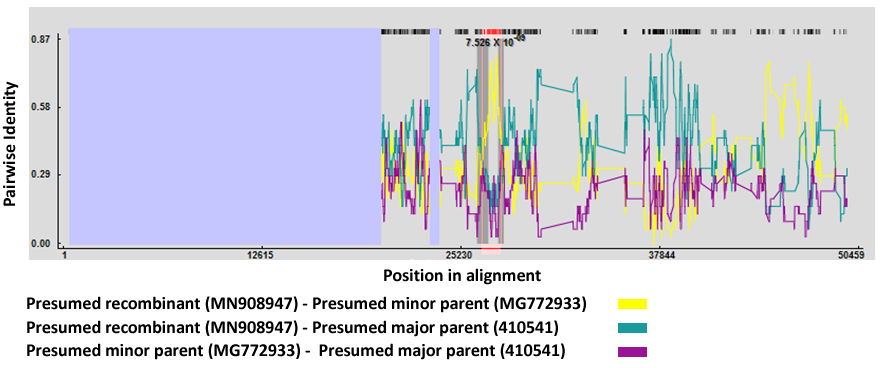


**B**


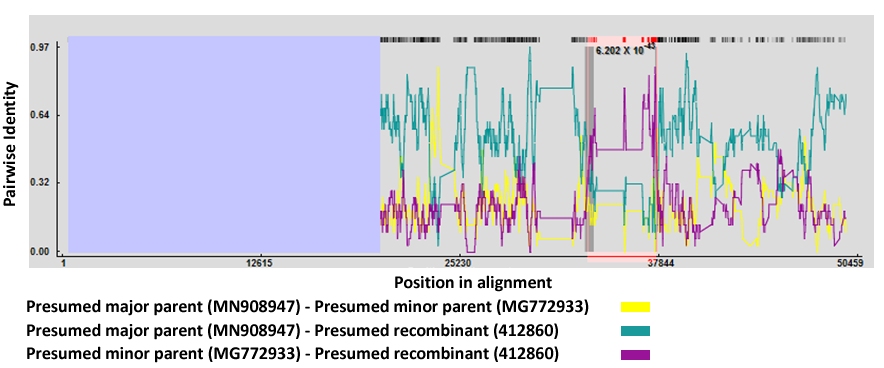


**C**


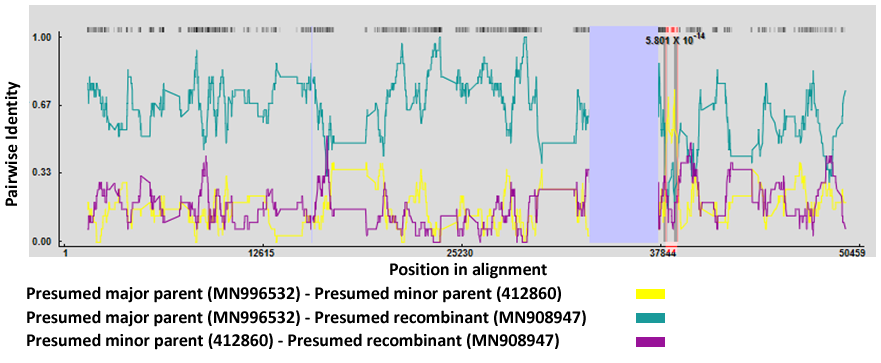


Figure S1. Pairwise identity plots extracted from RDP4. We manually revised the original snapshots to increase the legibility of the figures. The IDs of representative strains for the major parent, minor parent and recombinant are presented in small brackets. A) Recombination of a strain similar to Pangolin-CoV-2017 (410541), a strain similar to Bat-SL-CoV (MG772933) and ancestral strains of SARS-CoV-2, Bat-CoV-RaTG13 and Pangolin-CoV-2019 (MN908947). B) Recombination of a strain similar to SARS-CoV-2 (MN908947), a strain similar to Bat-SL-CoV (MG772933) and a strain similar to Pangolin-CoV-2019 (412860). C) Recombination of a strain similar to Bat-CoV-RaTG13 (MN996532), a strain similar to Pangolin-CoV-2019 (412860) and a strain similar to SARS-CoV-2 (MN908947). In each diagram, major parents and minor parents refer to the descriptions in Table 1. The putative recombination regions are marked in red.


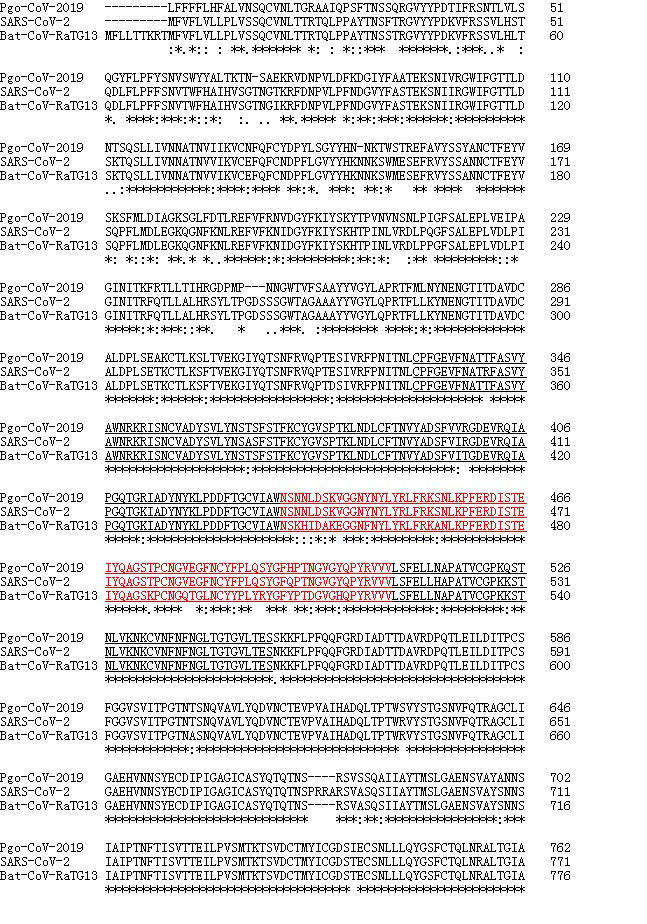

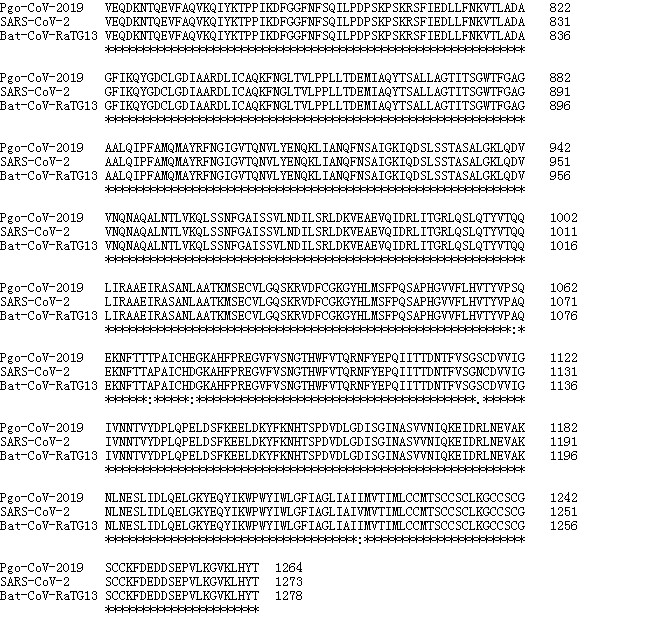


Figure S2. Multiple sequence alignment of S proteins in Pgo-CoV-2019, SARS-CoV-2 and Bat-CoV-RaTG13. The underlined letters indicate RBD, and those marked in red indicate the recombination region.


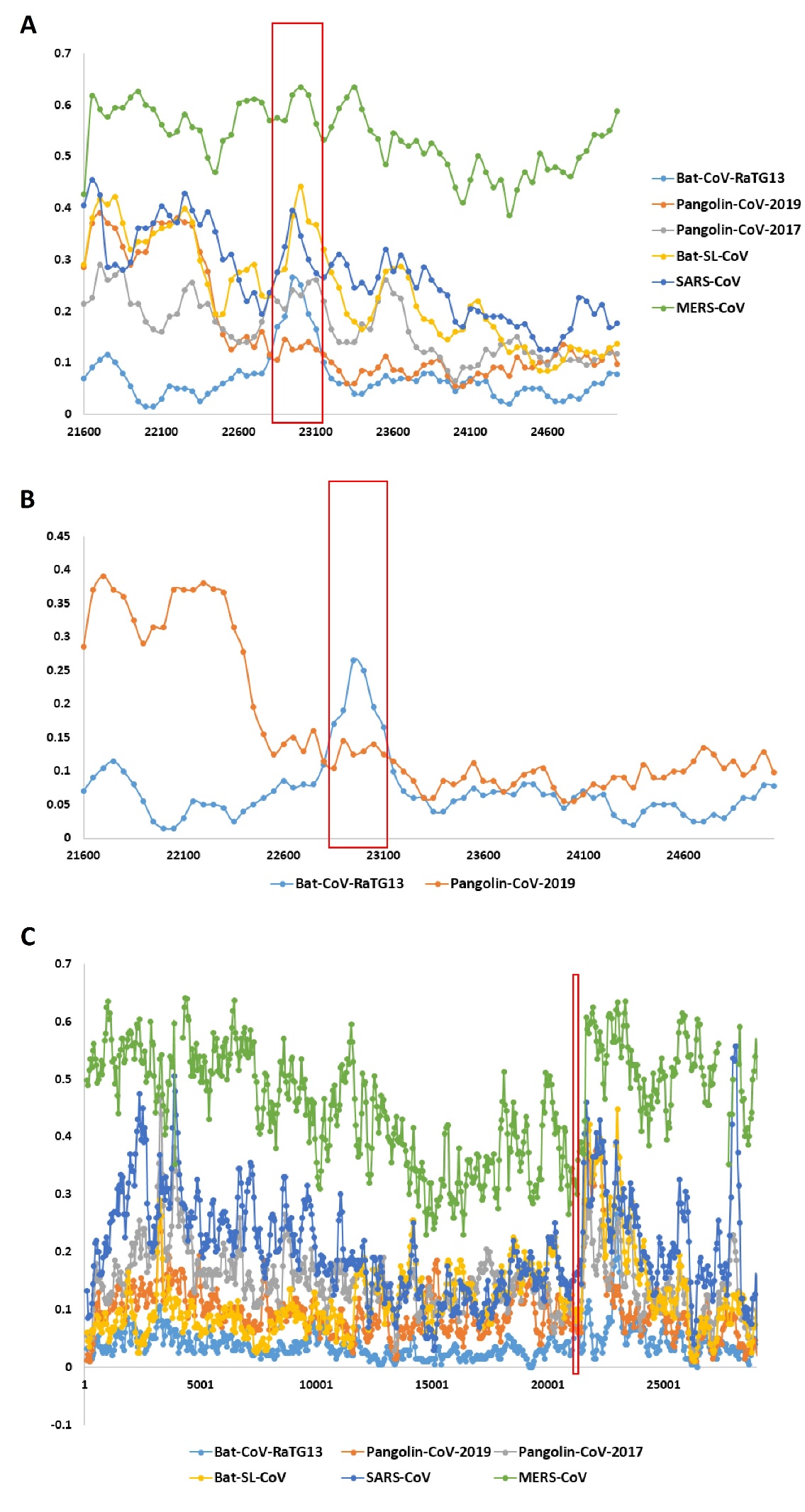


Figure S3. Sliding window analysis of nucleotide differences between SARS-CoV-2 and other coronaviruses proximal to SARS-CoV-2 in the phylogenetic tree shown in Figure 1A. The red rectangle denotes the location of the putative recombination region in the S protein. The Y axis is the percentage of dissimilar nucleotides in a window (200 bp). The sliding step size is 50 bp. A and C show comparisons of SARS-CoV-2 and 6 coronaviruses, while B shows SARS-CoV-2 vs Bat-CoV-RaTG13 and SARS-CoV-2 vs Pangolin-CoV-2019. A and B are analyses of the region from 21500 bp to 25000 bp. C is that of the whole coronavirus genome.


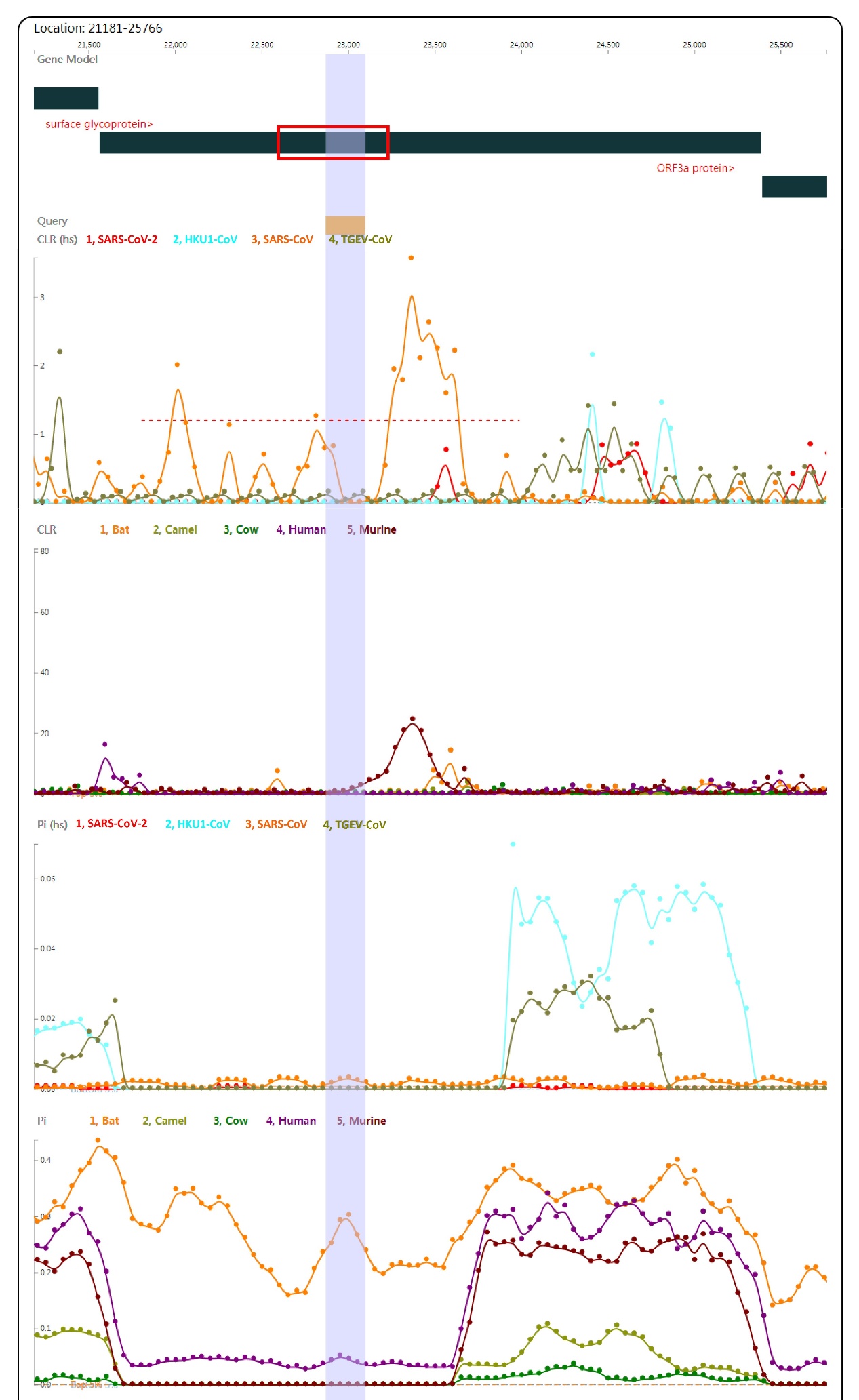


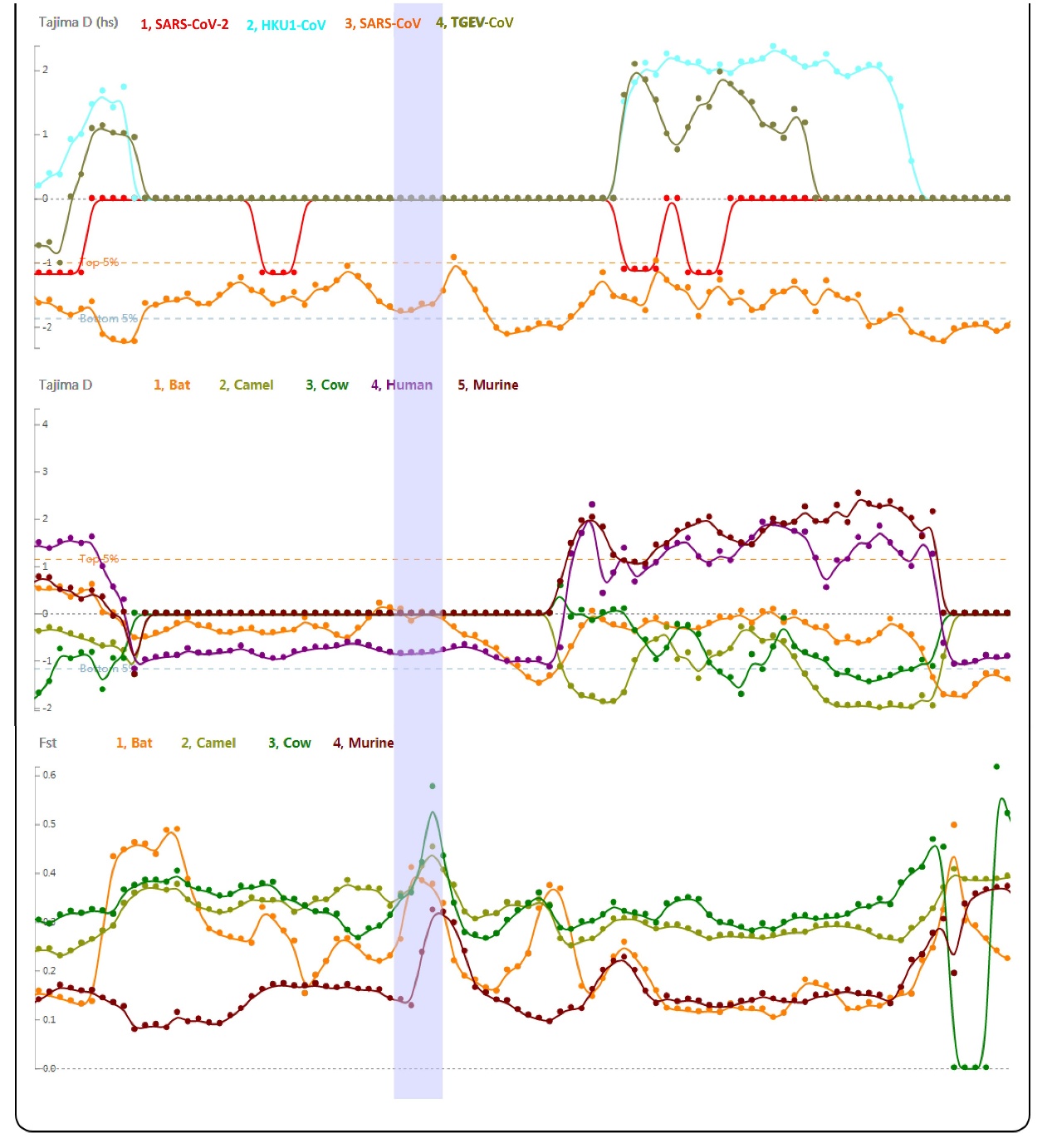


Figure S4. A snapshot of the SARS-CoV-2 (MN908947) spike glycoprotein in CoVdb’s genome browser, with the recombination region marked by a light blue bar and the RBD (331 AA to 550 AA in the spike glycoprotein) marked by a red rectangle. Tracks of population genetic tests are listed following gene segments. “CLR (hs)” was calculated for strains of SARS-CoV-2, HKU1-CoV, SARS-CoV and TGEV-CoV. “CLR” represents the CLR value within human, bat, camel, cow or murine isolates. The same is true for Pi and Tajima’s D. Fst was calculated between human and nonhuman (bat, camel, cow or murine) isolates. In the tracks of “CLR (hs)”, the top 0.05 cutoff with the whole genome as the background is indicated by a red dashed line.

**A**


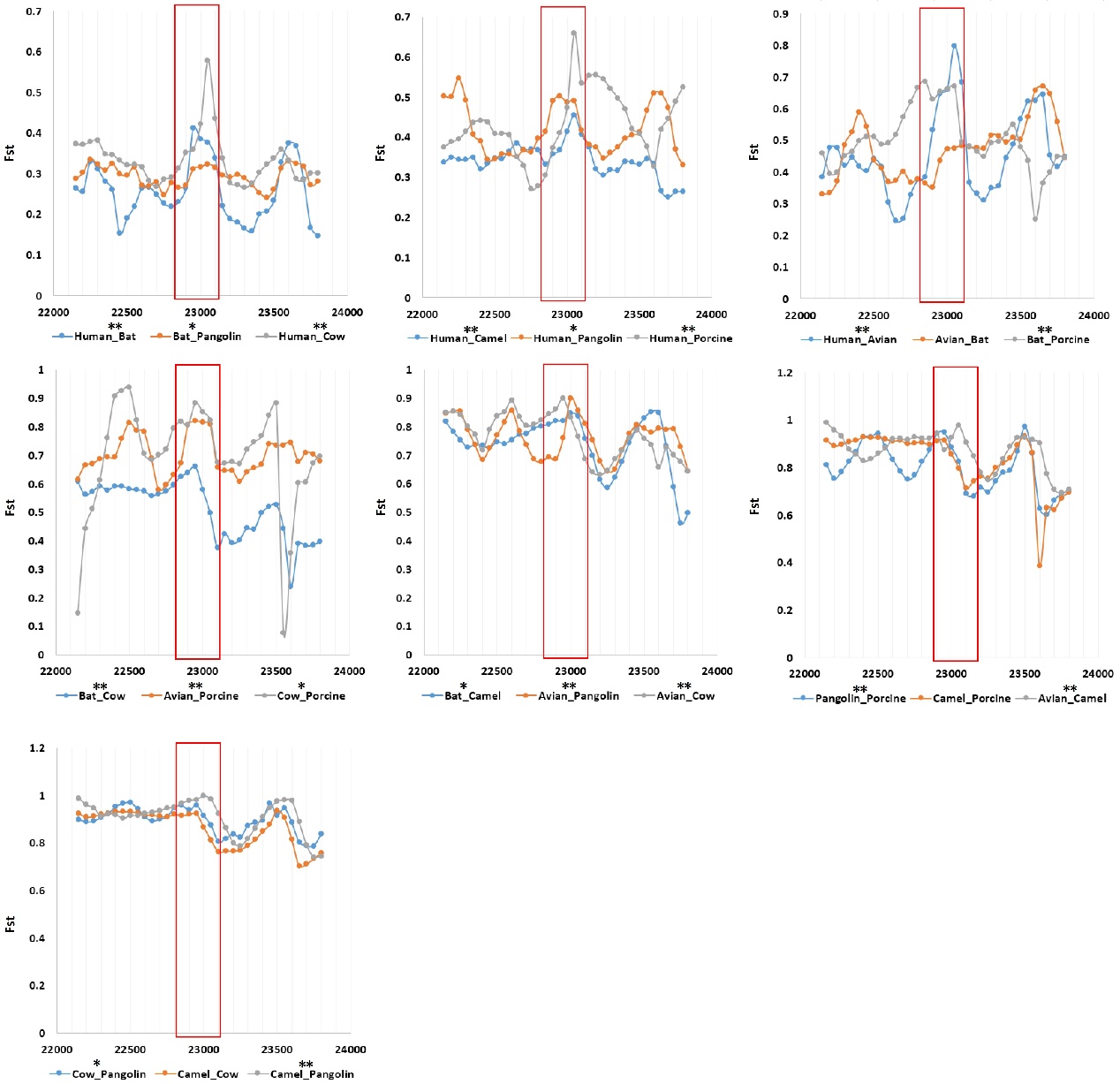


**B
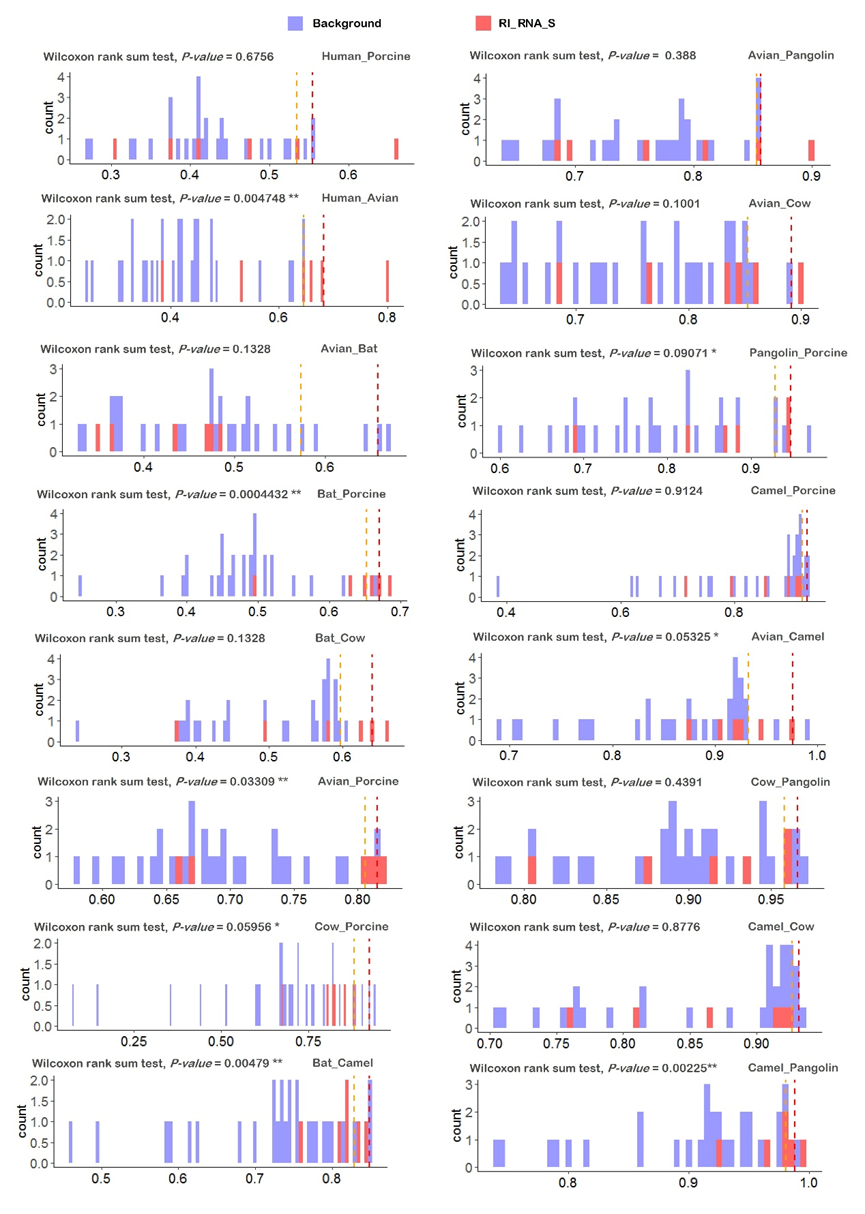
**

Figure S5. A) Snapshots of sliding window analysis of Fst in RI_RNA_S between coronaviruses isolated from pairs of hosts, including human, avian, pangolin, porcine, bat, camel and cow hosts. For convenience, only three tracks are listed in a subfigure. The peaks with values higher than the 0.1 threshold in the nearby region are marked by ‘*’ (weak significance) above the legends. Those with values higher than the 0.05 threshold in the nearby region are marked by ‘**’ (significant). B) Comparison of the distributions of Fst in RI_RNA_S and the nearby region (background), from Human_Porcine to Camel_Pangolin. The legends in B follow those in Figure 2D. The other 5 pairs of hosts being compared are shown in Figure 2D.


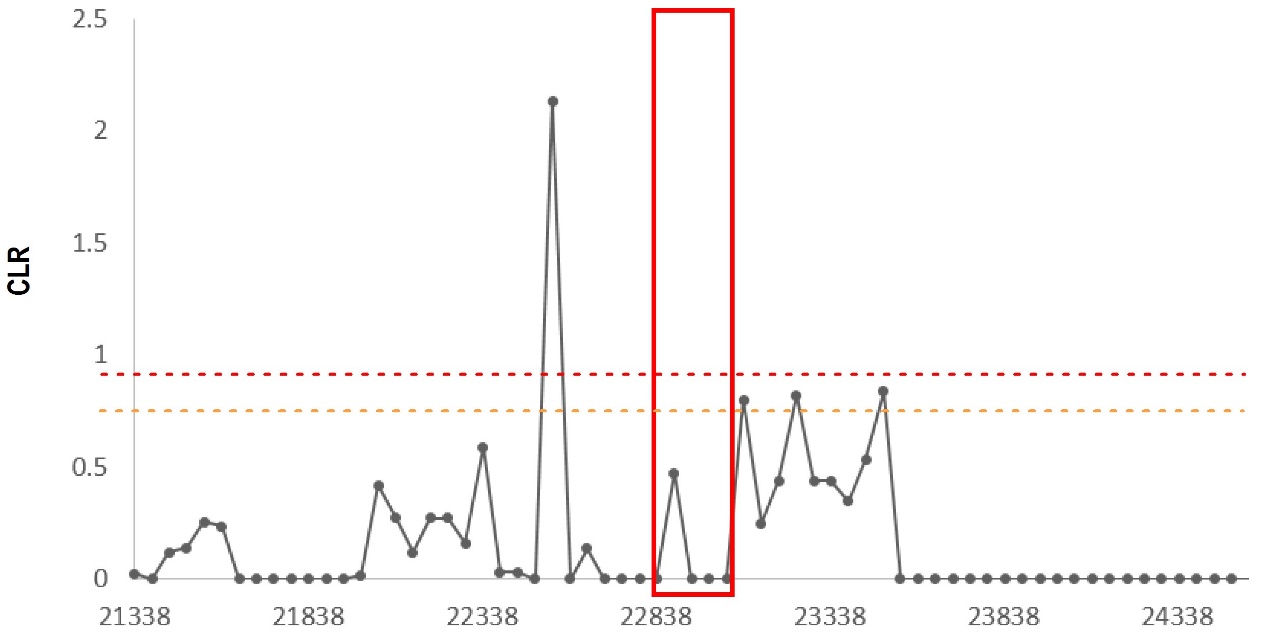


Figure S6. Sliding window analysis of the CLR, with RI_RNA_S marked by a red rectangle. The result was generated using SARS-CoV-2 strains collected in March. The top 0.05 cutoff of the values in the nearby region (21000 bp to 25000 bp) is denoted by an orange dashed line, while that in the whole genome is denoted by a red dashed line.

**A**


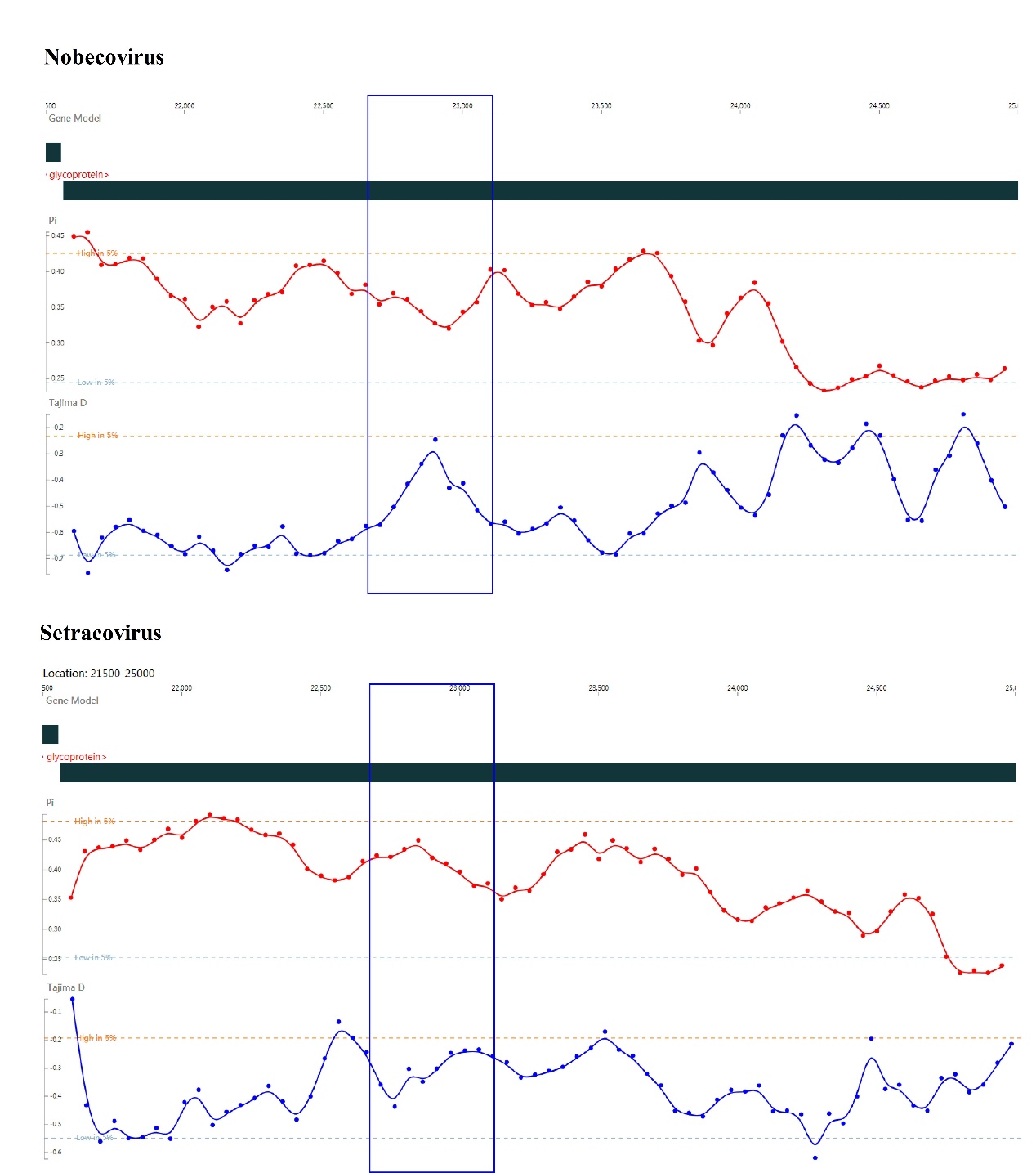


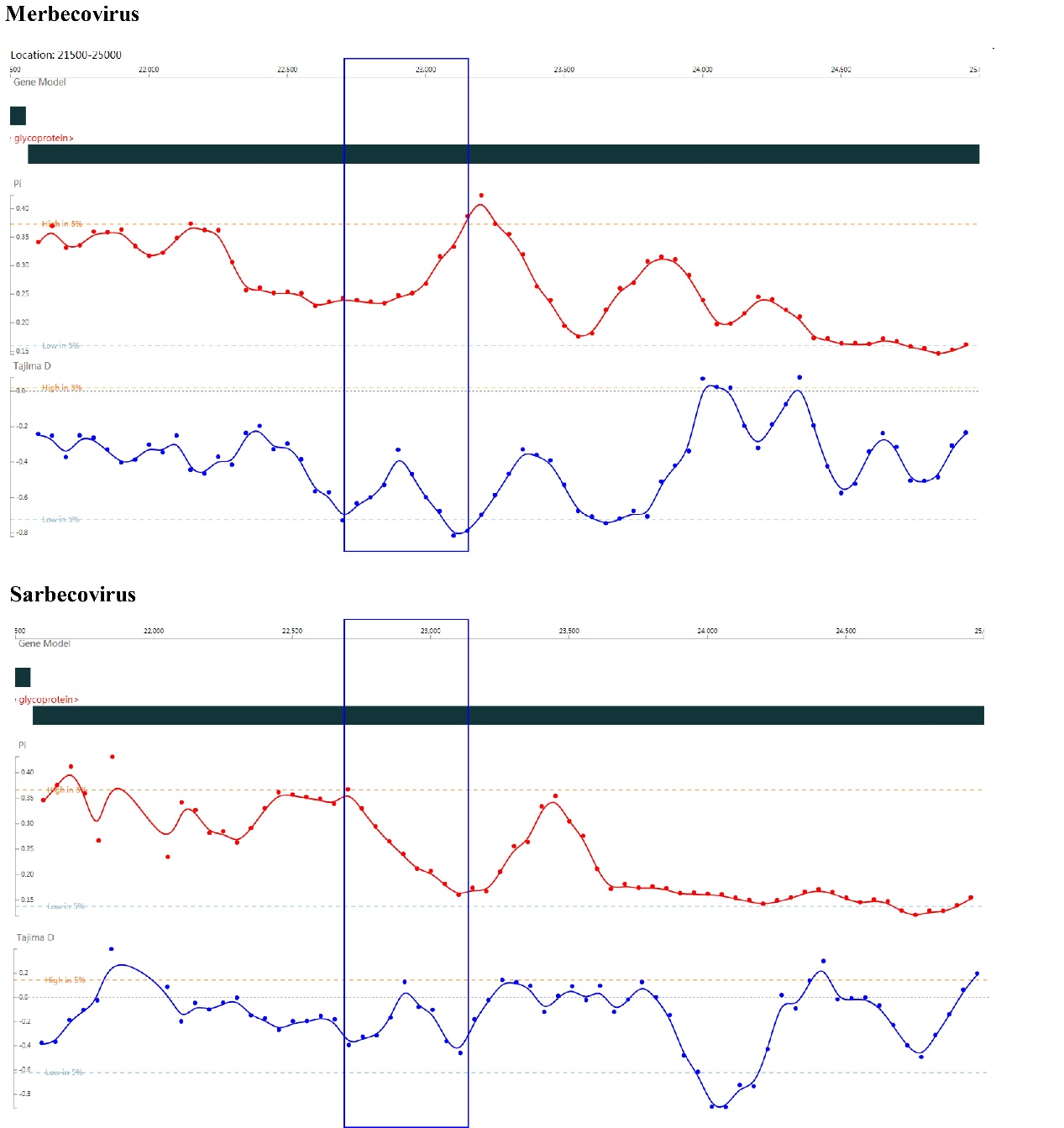


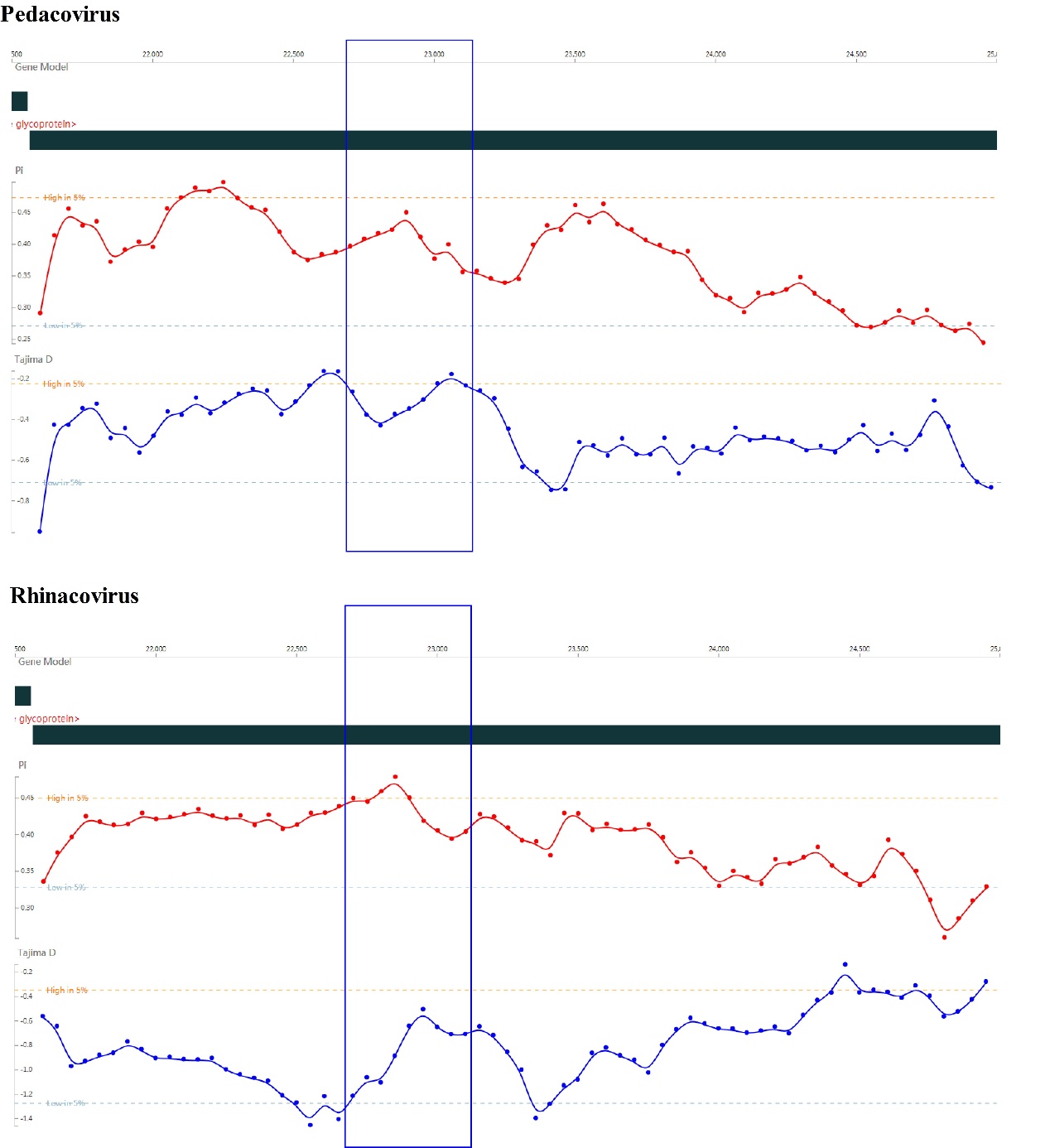


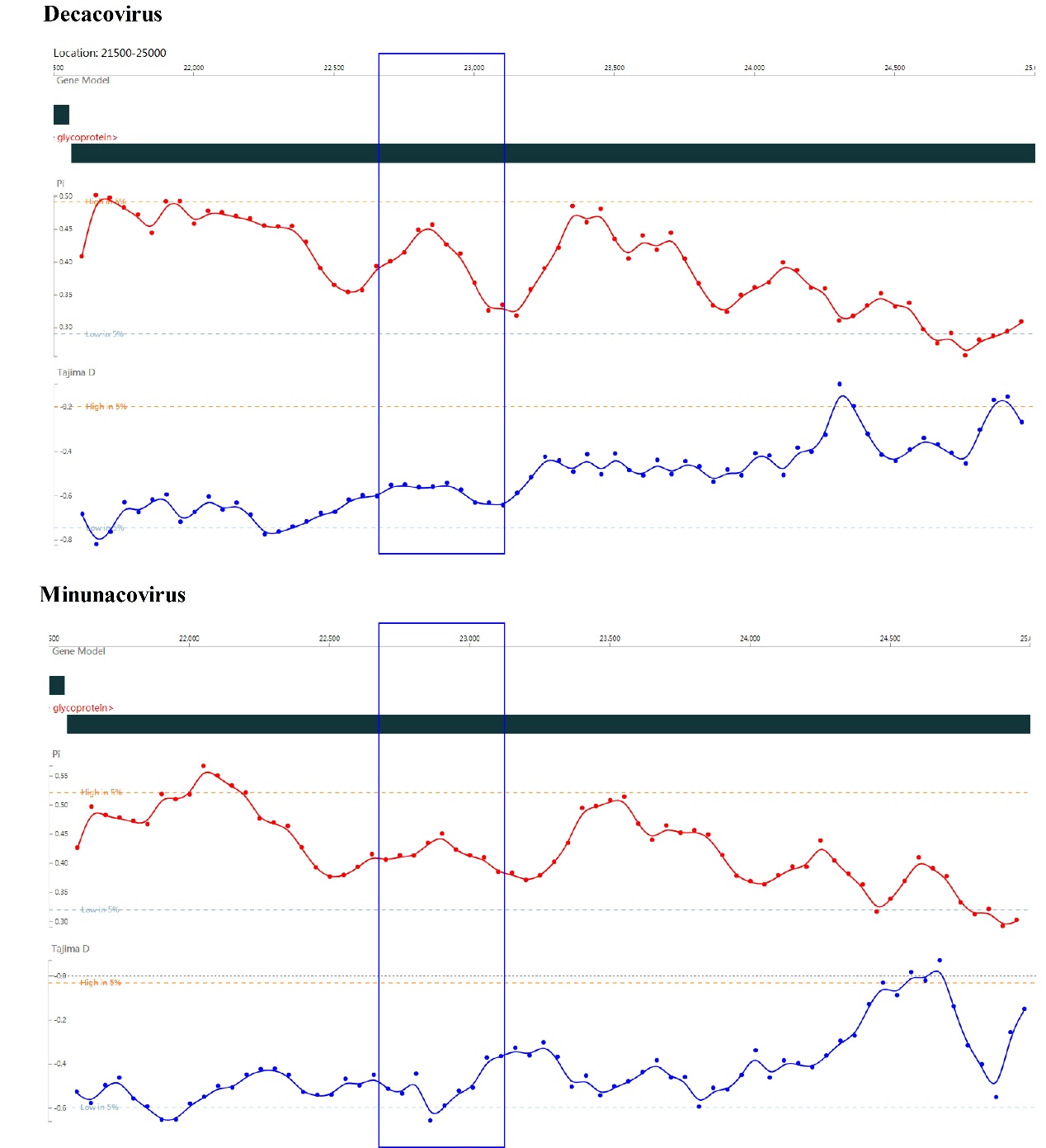


**B**


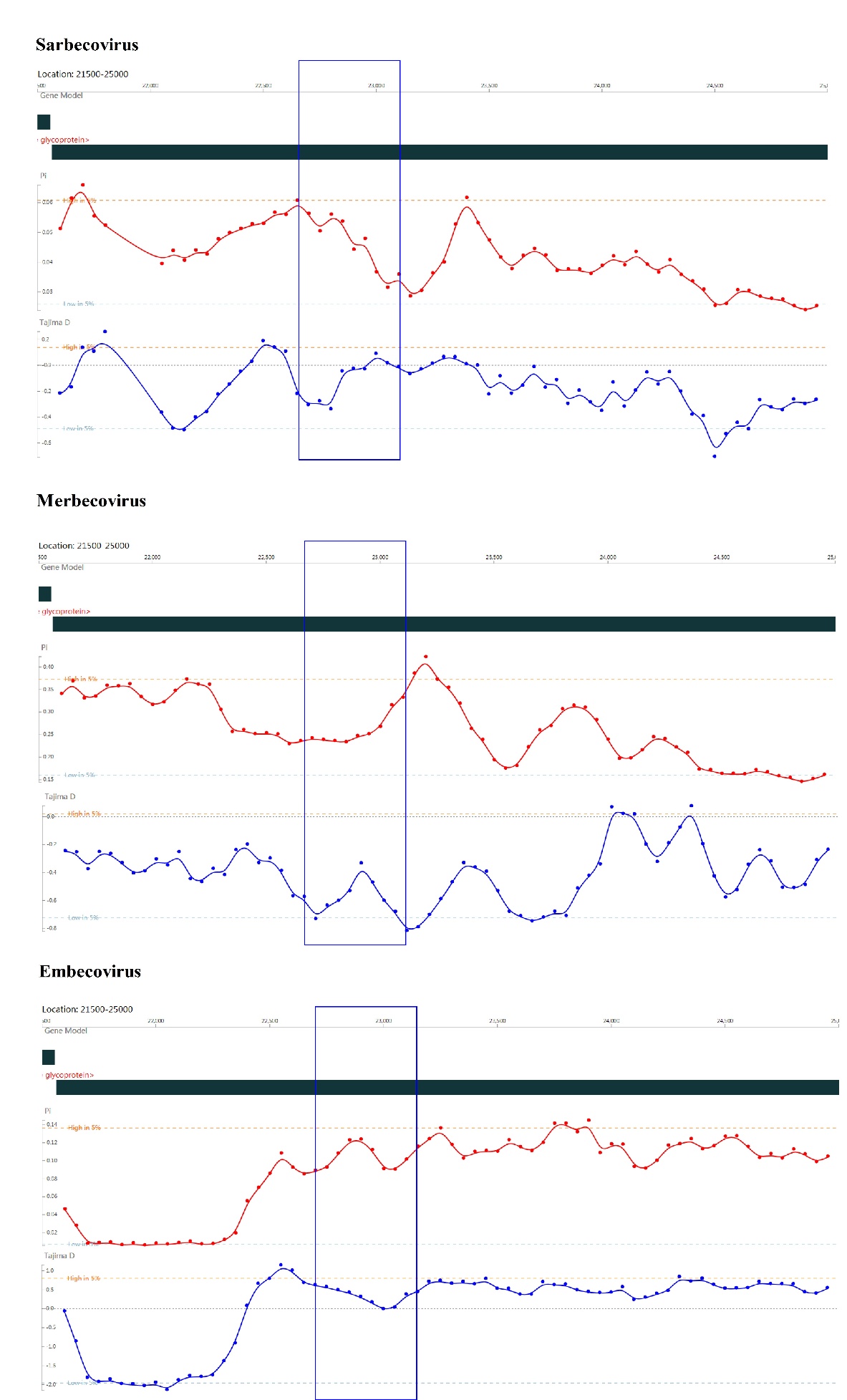


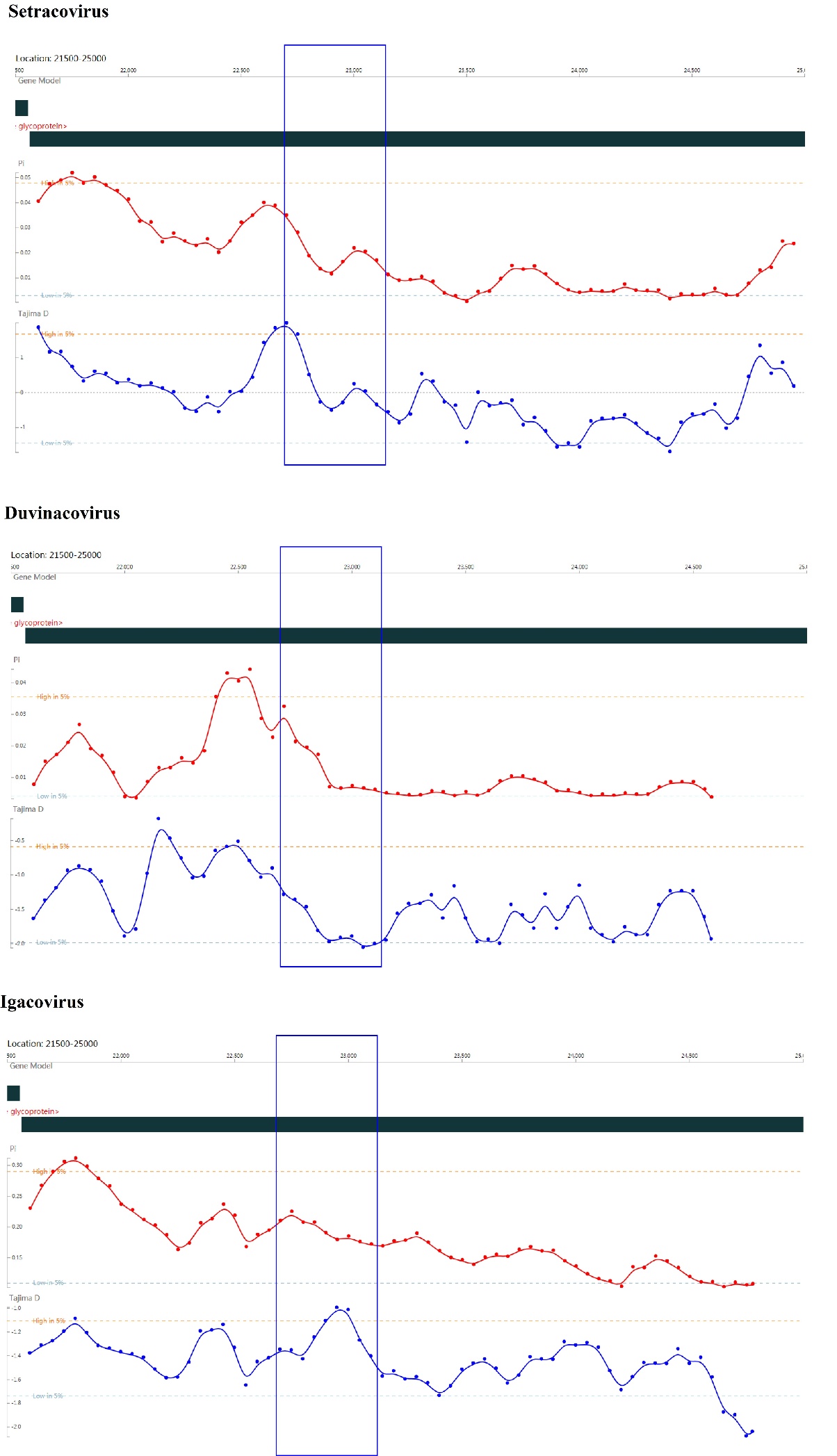


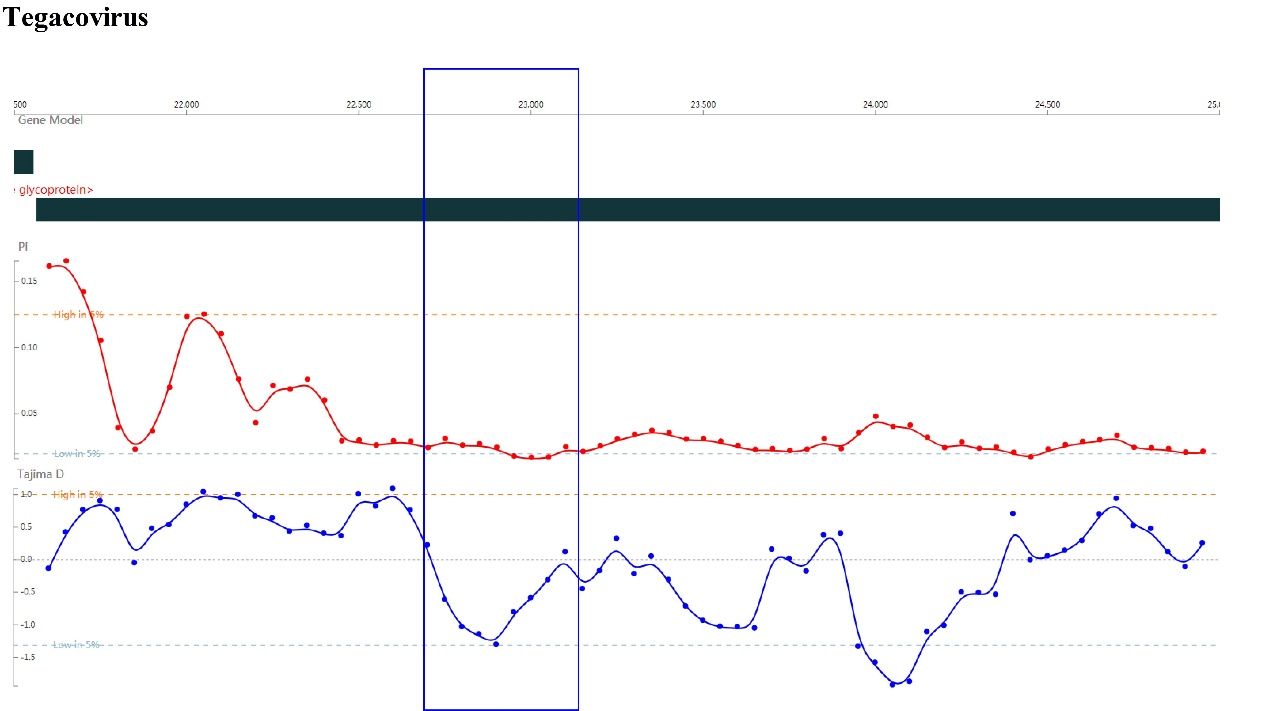


Figure S7. A. Snapshots of sliding window analyses of Pi and Tajima’s D for bat isolates of different subdivisions. We performed analyses of subdivisions with more than 10 strains. B. Snapshots of sliding window analyses of Pi and Tajima’s D for human isolates belonging to different subdivisions. The blue rectangle denotes the position of RI_RNA_S. In each figure, the dotted orange line is the top 5% cutoff within the viewed region. In A, Rhinacovirus has a Pi peak above the top 5% line.

**A**

**
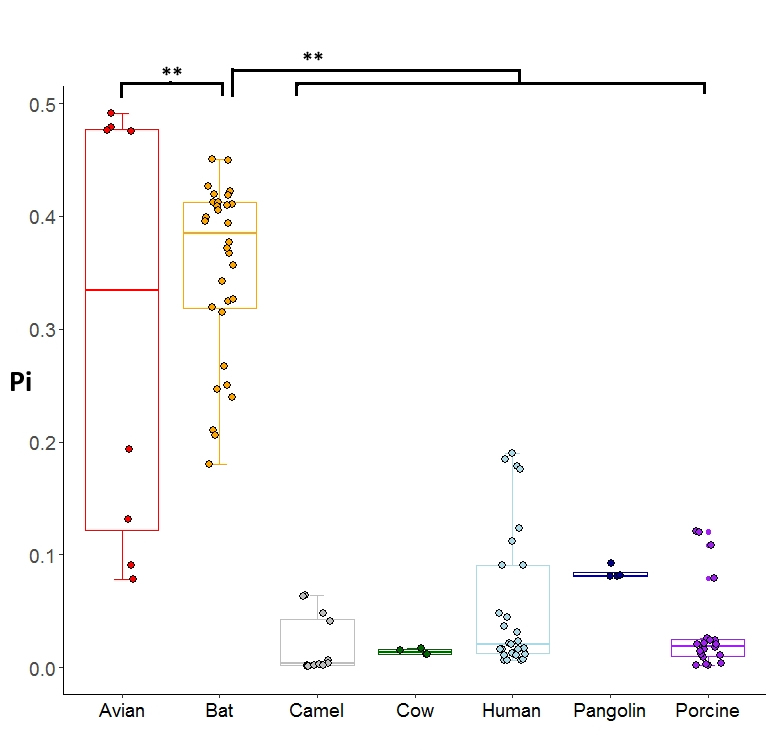
**

**B**

**
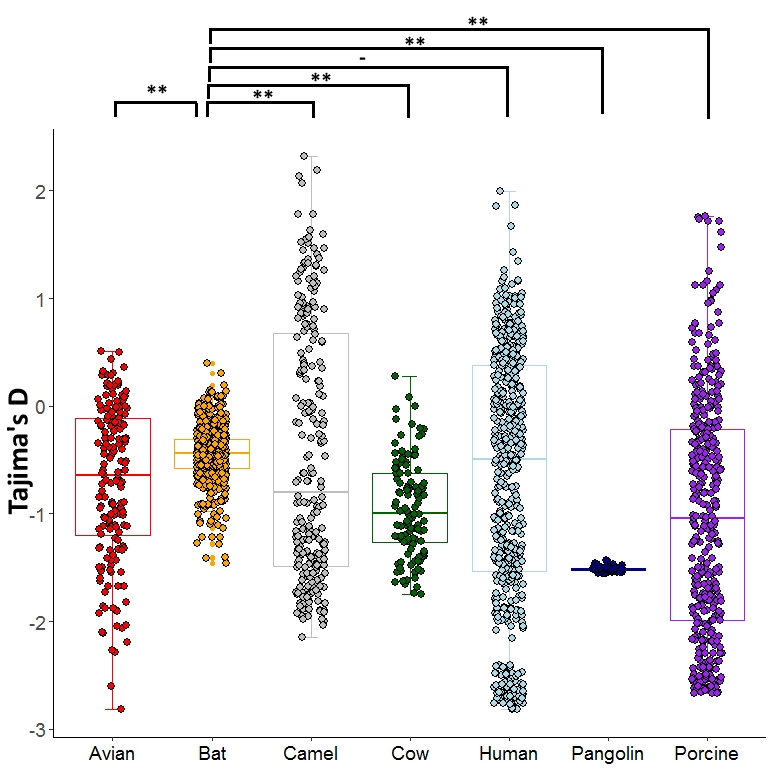
**

**C**

**
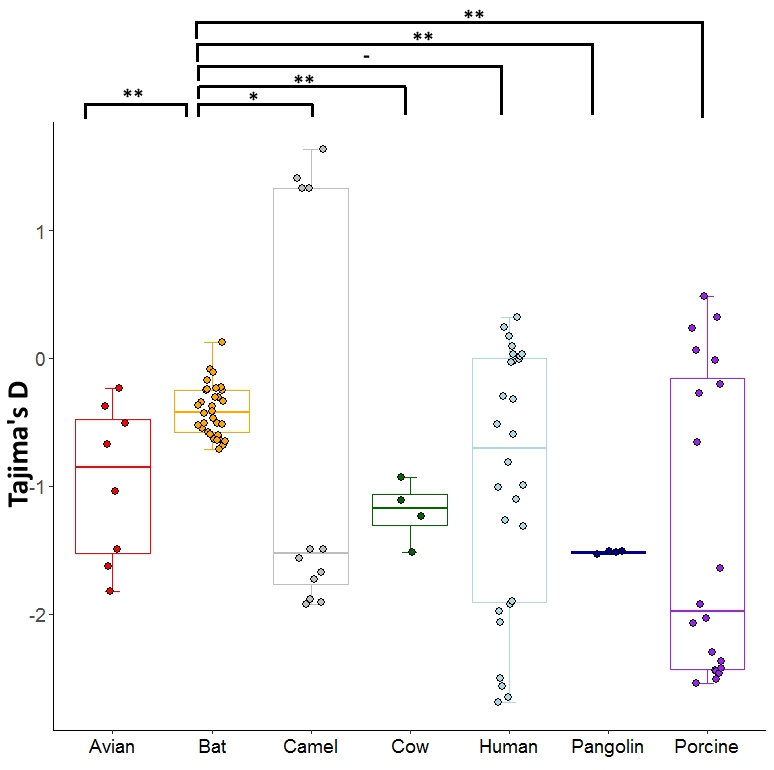
**

Figure S8. Comparison of Pi or Tajima’s D of coronavirus clades from 7 different hosts. The values of Pi or Tajima’s D were calculated with a window size of 200 bp and sliding step size of 50 bp. A is the comparison of Pi values in RI_RNA_S. B is the comparison of Tajima’s D in the region from 21500 bp to 25000 bp covering RI_RNA_S. C is the comparison of Tajima’s D in RI_RNA_S. ‘*’ denotes weak statistical significance (P-value<0.1, Wilcox rank sum test). ‘**’ denotes statistical significance (P-value<0.05, Wilcoxon rank sum test). ‘-‘ denotes no statistical significance.


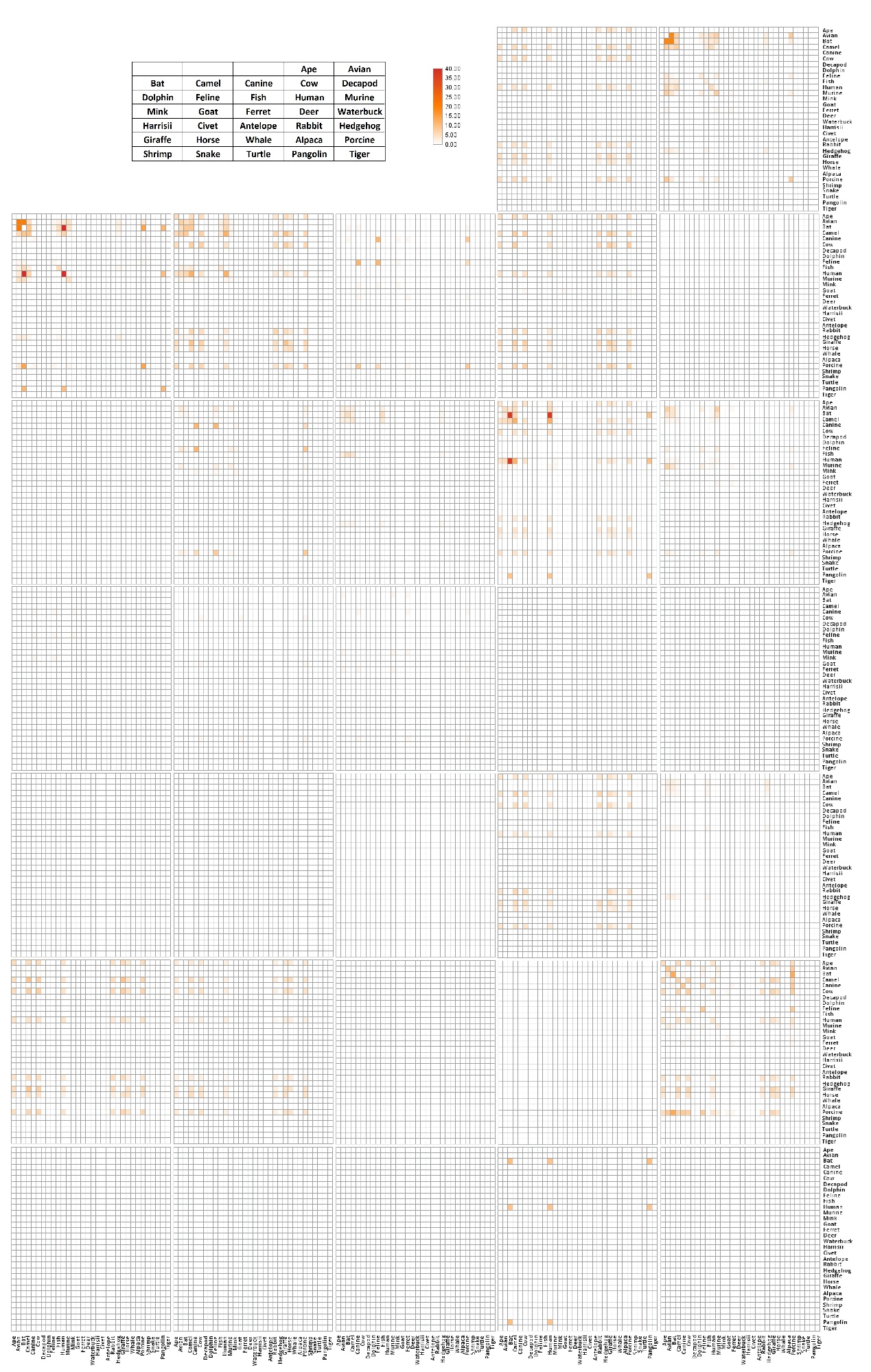


Figure S9. A list of heatmaps showing the numbers of independent recombination events that occurred in coronaviruses isolated from triplets of hosts. One host in the triplet is shown in the table in the top left corner. The other two hosts are listed on the x and y axes of each heatmap. We excluded recombination events that occurred between coronaviruses belonging to the same host. Their numbers were manually set to zero.


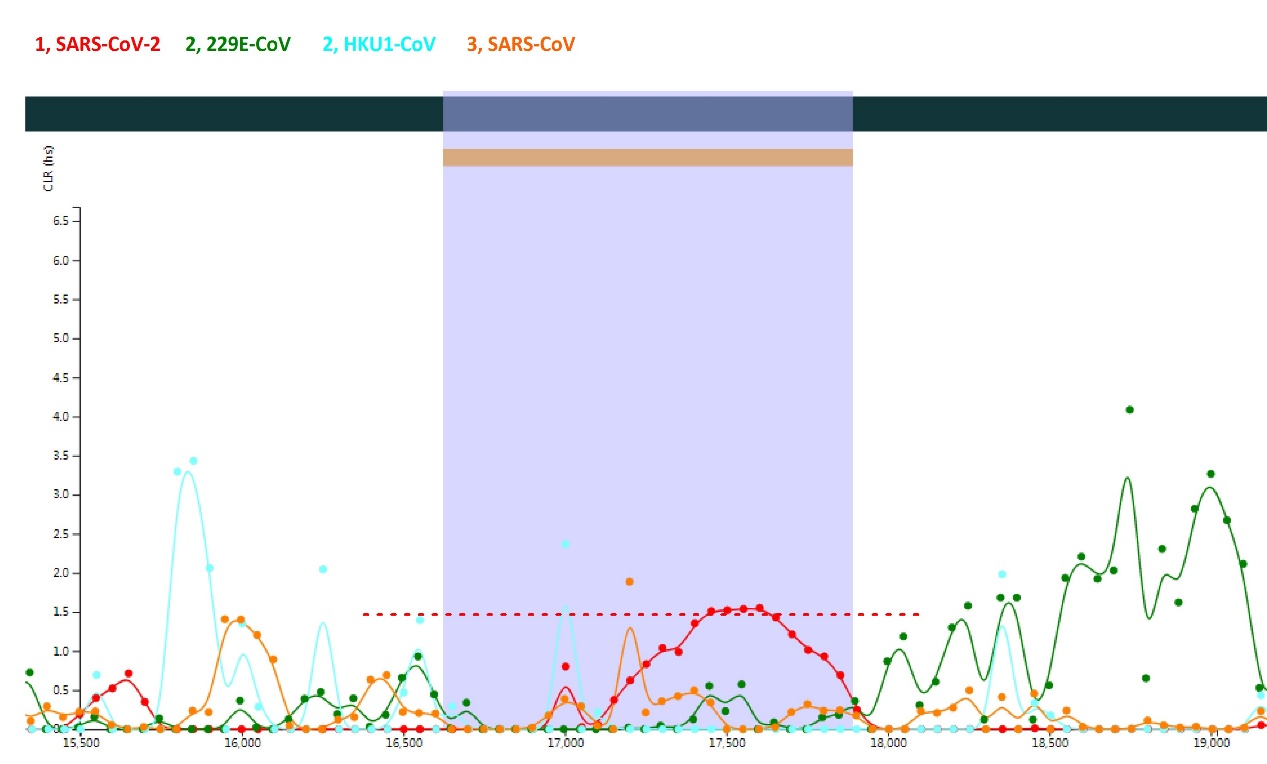


Figure S10. A snapshot of the genome of SARS-CoV-2 (MN908947) in the genome browser (CoVdb), with RI_RNA_ORF1 marked by a light blue bar. Legends for the tracks of population genetic analysis follow those in Figure S4. The red dashed line is the top 0.05 threshold of the CLR for SARS-CoV-2 in the nearby region (from 15500 bp to 19500 bp). Pairs of distributions in RI_RNA_ORF1 and the flanking region were compared by a Wilcoxon rank sum test, and the *P-value* was 9.997e-08.


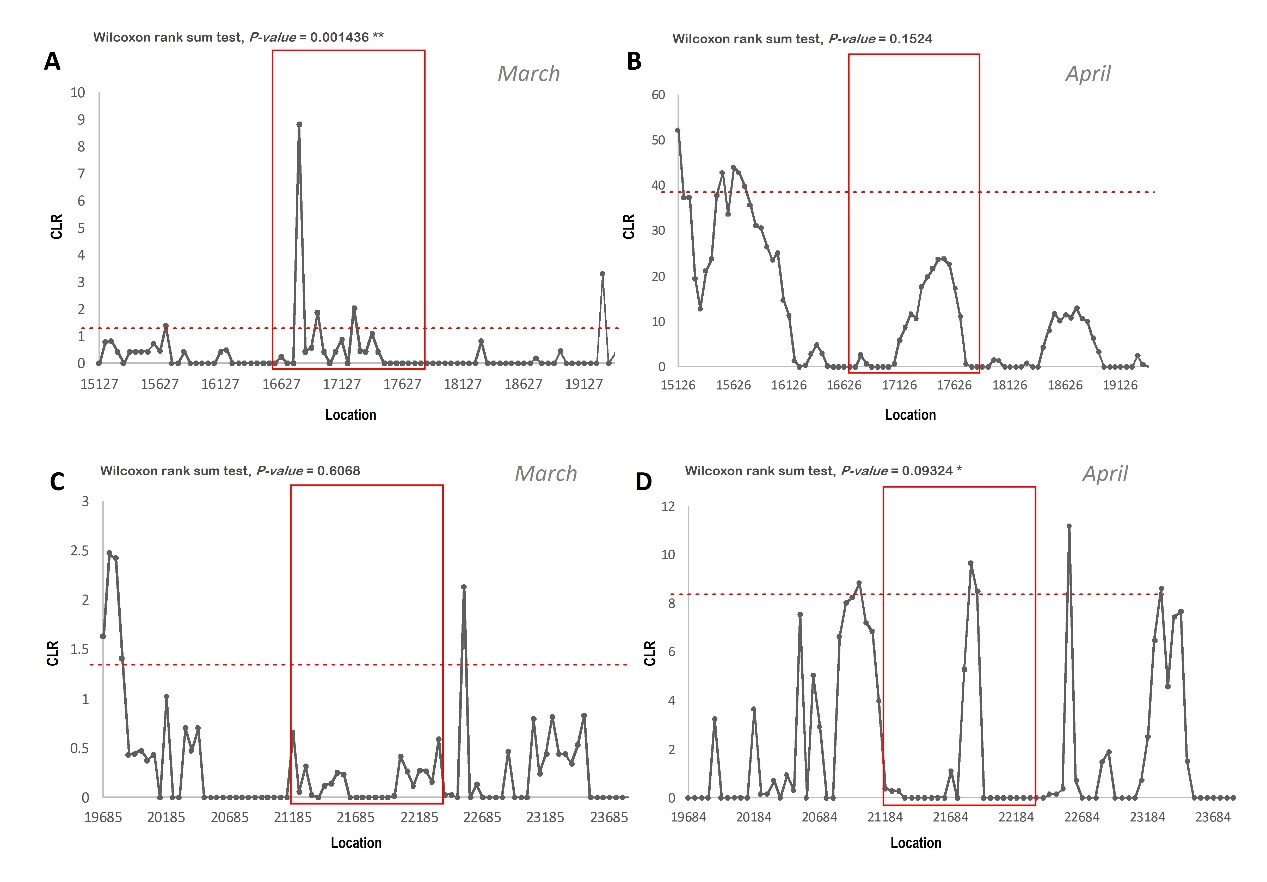


Figure S11. Sliding window analysis of CLRs, with the recombination region marked by a red rectangle. A and B are those in RI_RNA_ORF1. C and D are those in RI_RNA_Boundary. A and C were generated using all reported SARS-CoV-2 strains collected in March, while B and D were generated using all reported SARS-CoV-2 strains collected in April. The red dashed line in each subfigure is the top 0.05 threshold of the CLRs for SARS-CoV-2 in the nearby region, extended by 1000 bp flanking the target region. Pairs of distributions in the recombination region and the flanking region were tested by a Wilcoxon rank sum test, and a *P-value* is given.


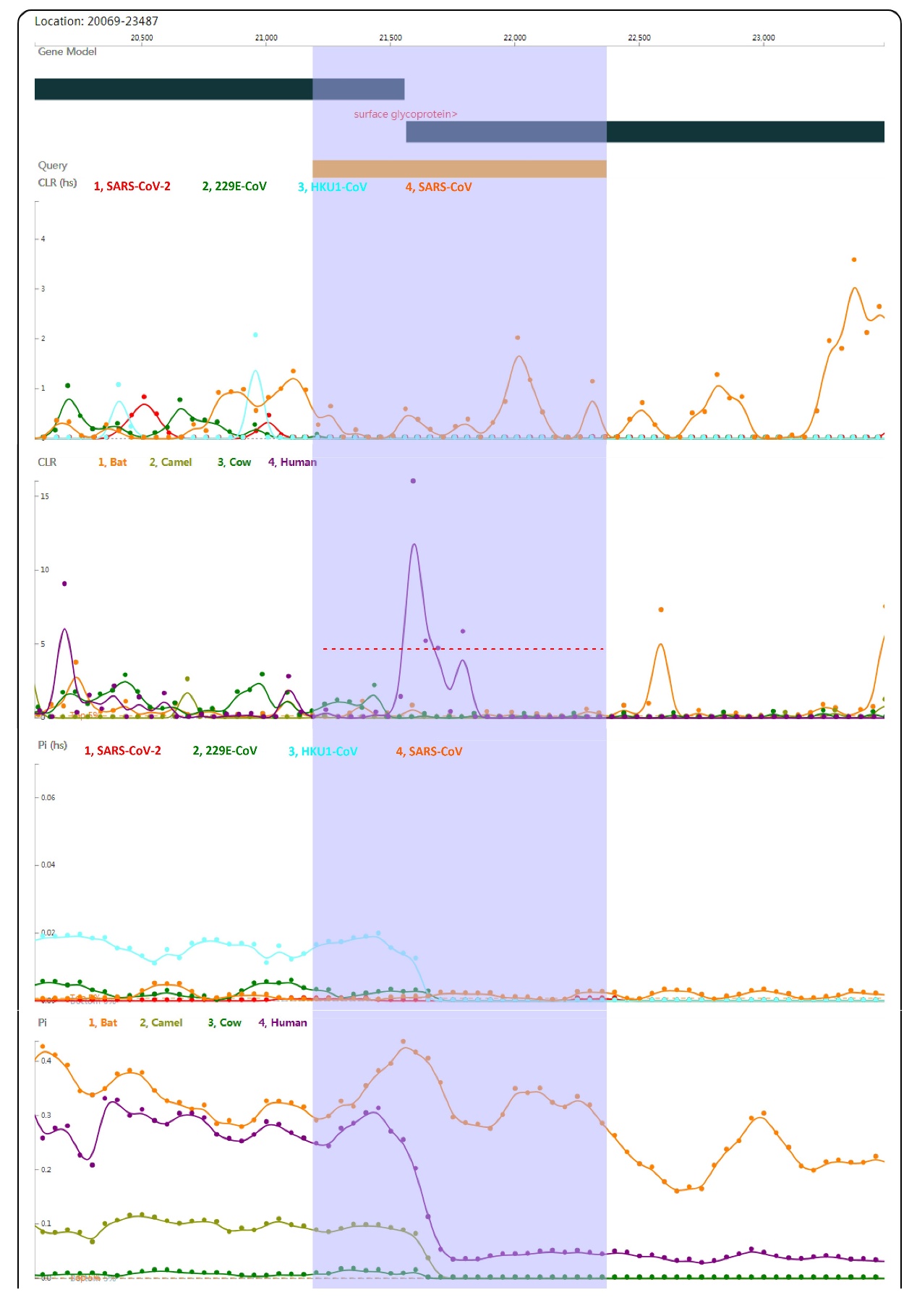

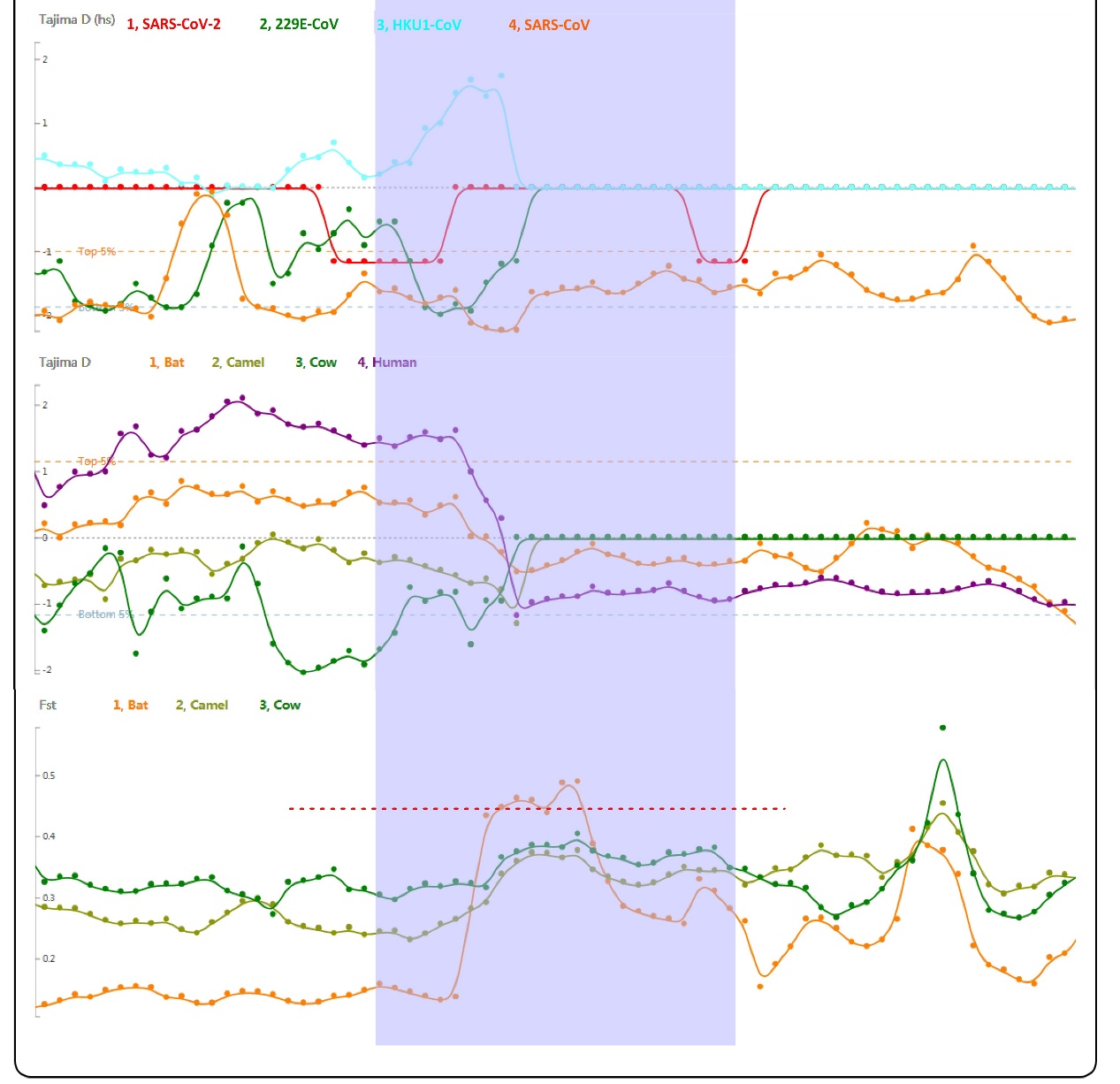


Figure S12. A snapshot of the SARS-CoV-2 genome (MN908947) in the genome browser (CoVdb), with RI_RNA_Boundary marked by a light blue bar. Legends for the tracks of population genetic tests follow those in Figure S4. The red dashed lines in the tracks of CLR and Fst values are the top 0.05 thresholds in the nearby region, extended by 1000 bp flanking the target region.


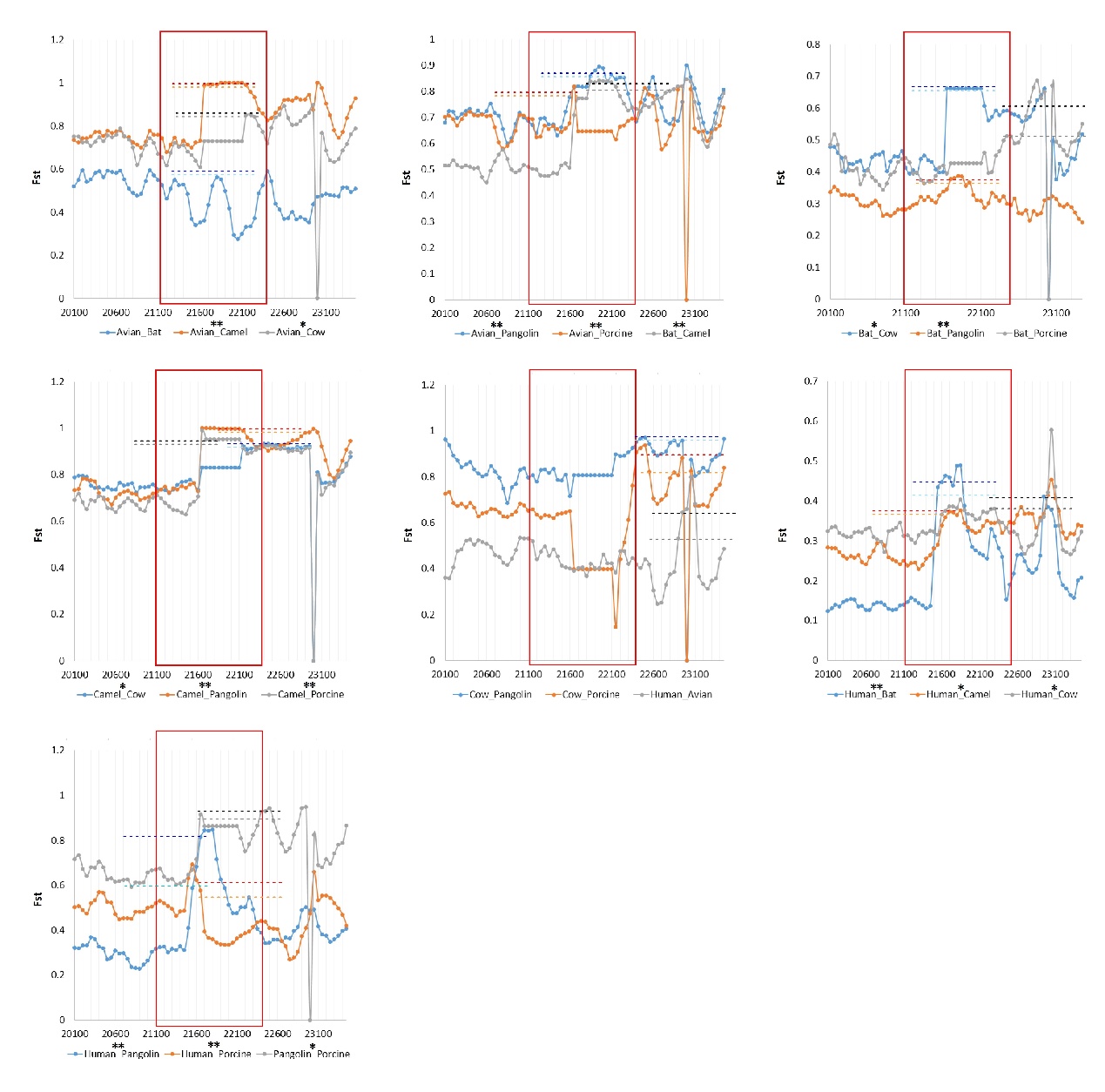


Figure S13. Snapshots of sliding window analysis of Fst in RI_RNA_Boundary between coronaviruses isolated from pairs of hosts, including human, avian, pangolin, porcine, bat, camel and cow hosts. The legends follow those in Figure S5A. The dashed lines represent the top 0.05 (dark) and 0.1 (light) thresholds in the nearby region. The dashed lines in dark and light blue correspond to the blue tracks, those in red and orange correspond to the orange tracks, and those in black and gray correspond to the gray tracks.
